# Strategies for improving health care seeking for maternal and newborn illnesses in low- and middle-income countries: a systematic review and meta-analysis

**DOI:** 10.3402/gha.v9.31408

**Published:** 2016-05-10

**Authors:** Zohra S. Lassi, Philippa F. Middleton, Zulfiqar A. Bhutta, Caroline Crowther

**Affiliations:** 1 Australian Research Centre for Health of Women and Babies, Robinson Research Institute, School of Paediatrics and Reproductive Health, The University of Adelaide, Adelaide, Australia; 2 Women's and Children's Health Research Institute, The University of Adelaide, Adelaide, Australia; 3 Centre for Global Child Health, The Hospital for Sick Children, Toronto, Canada; 4 Centre of Excellence for Women and Child Health, The Aga Khan University, Karachi, Pakistan; 5 Liggins Institute, University of Auckland, Auckland, New Zealand

**Keywords:** health care seeking, maternal health, neonatal health, neonatal mortality, perinatal mortality, developing countries, low- and middle-income countries

## Abstract

**Background:**

Lack of appropriate health care seeking for ill mothers and neonates contributes to high mortality rates. A major challenge is the appropriate mix of strategies for creating demand as well as provision of services.

**Design:**

Systematic review and meta-analysis of experimental studies (last search: Jan 2015) to assess the impact of different strategies to improve maternal and neonatal health care seeking in low- and middle-income countries (LMIC).

**Results:**

Fifty-eight experimental [randomized controlled trials (RCTs), non-RCTs, and before-after studies] with 310,652 participants met the inclusion criteria. Meta-analyses from 29 RCTs with a range of different interventions (e.g. mobilization, home visitation) indicated significant improvement in health care seeking for neonatal illnesses when compared with standard/no care [risk ratio (RR) 1.40; 95 confidence interval (CI): 1.17–1.68, 9 studies, *n=*30,572], whereas, no impact was seen on health care seeking for maternal illnesses (RR 1.06; 95% CI: 0.92–1.22, 5 studies, *n=*15,828). These interventions had a significant impact on reducing stillbirths (RR 0.82; 95% CI: 0.73–0.93, 11 studies, *n=*176,683), perinatal deaths (RR 0.84; 95% CI: 0.77–0.90, 15 studies, *n=*279,618), and neonatal mortality (RR 0.80; 95% CI: 0.72–0.89, 20 studies, *n=*248,848). On GRADE approach, evidence was high quality except for the outcome of maternal health care seeking, which was moderate.

**Conclusions:**

Community-based interventions integrating strategies such as home visiting and counseling can help to reduce fetal and neonatal mortality in LMIC.

## Background

Globally, deaths of mothers and newborn babies are far too high. Every year an estimated 289,000 mothers and 2.62 million newborns die globally (1, 2). Complications during pregnancy and childbirth often lead to emergency situations, with a slim window of time to intervene. Maternal health complications contribute to 1.5 million early neonatal deaths and 1.4 million stillbirths, suggesting that there is a major gap requiring intervention around the time of birth and in the early postnatal period, a time when mothers and babies are most at risk (3). Worldwide, 50 million births take place at home without a skilled birth attendant (SBA) (4). Skilled attendance at birth remains unacceptably low in sub-Saharan Africa and Southern Asia and there are further wide disparities within countries, across socio-economic status, geographic location, and educational status (5).

With 99% of maternal, newborn, and child deaths occurring in low- and middle-income countries (LMICs), increasing health resources and appropriate intervention in these countries is an urgent priority and global responsibility for reducing the burden of maternal and child mortality (6, 7). Antenatal care provides an opportunity to not only detect potential complications but also to prevent them. Birth preparedness – an easy to deliver and inexpensive intervention – can avert the brunt of maternal and perinatal mortalities. It includes different interventions such as identifying SBAs, the closest appropriate health facility, and sometimes funds for emergency transportation and consultation, all of which can reduce delays in obtaining care (8). During the last decade a number of systematic reviews have been published which have assessed interventions for improving maternal and newborn health (9–38). However, none of these have specifically focused on strategies to improve maternal and newborn health care seeking, the aim of this systematic review and meta-analysis.

## Methods

All experimental studies from LMICs that assessed the health care seeking behavior or pattern for maternal and newborn health care and illnesses were included. The population for this review included pregnant women at any gestation, postpartum women up to 6 weeks after giving birth, and neonates less than 28 days of life. We included studies that provided information and education for empowerment and change in the form of group meetings or individual one-to-one counseling at home or at primary health care facilities and compared them with standard/no care. The primary outcomes assessed were health care seeking for maternal and newborn illnesses. The secondary outcomes included maternal, neonatal, and perinatal mortality, stillbirths, (Panel 1) and maternal and newborn care outcomes, such as antenatal care, institutional births, and early initiation of breastfeeding.

**Panel 1:** Definitions.

•	Neonatal death: death of a live born infant within 28 completed days of birth.
•	Early neonatal death: deaths arising within 6 completed days of birth.
•	Late neonatal death: deaths arising from 7 to 28 completed days of birth.
•	*Stillbirth: baby born with no signs of life at or after 28 weeks’ gestation.
•	*Perinatal death: a stillbirth or early neonatal death.
•	Maternal death: death of a woman while pregnant or within 42 days of cessation of pregnancy from any cause related to the pregnancy or its management, but not from accidental causes.

* Stillbirths and perinatal deaths were defined differently in few studies. We considered author's definitions.

The protocol for this systematic review and meta-analysis was registered with PROSPERO 2012:CRD42012003236 (www.metaxis.com/prospero/full_doc.asp?RecordID=3236). This review was conducted in accordance with methods of the Cochrane Collaboration (39). Ovid platform was used to search PubMed, MEDLINE, and EMBASE; Popline, the Cochrane Library, and Google Scholar were also searched up to 12 January2015. Search terms were a combination of and synonyms of (‘care seeking’ OR ‘care-seeking’ OR ‘health care’ OR ‘health care seeking’ OR ‘community based intervention*’ OR ‘community-based intervention*’) AND (mother* OR maternal OR women OR newborn* OR neonat*) used as medical subject headings and keyword terms in the title/abstract (Supplementary File 3). No language restrictions were applied. Grey literature (materials and research produced by organizations, [such as community health workers (CHWs) central, High Impact Practices etc.] outside of the traditional commercial or academic publishing and distribution channels) and reference lists of included studies were also searched to identify studies.

ZSL and PM independently reviewed the retrieved articles in two stages; first assessing relevance from the title and abstract, and if relevance was still unclear, reading the full text. Any disagreement was referred to a third reviewer (CC and ZAB). Studies were analyzed according to their study design i.e. randomized (and cluster) controlled trials (RCTs), non-randomized controlled trials (non-RCTs), and before-after studies.

ZSL and PM extracted data independently from each included study. Study design, country of study, participants, intervention, comparison, and duration of intervention were recorded for each study. If information was missing, authors were contacted. The methodological quality of studies was evaluated using standardized forms. The quality of controlled trials was assessed according to Cochrane methods (40). Prospective studies were graded using the methods described by the Effective Practice, Organization and Communication Cochrane review group (EPOC 2009) (41).

We performed statistical analysis of RCTs, non-RCTs, and before-after studies using the Review Manager software (42). For dichotomous data, we presented results as summary risk ratio (RR) and for continuous data we used mean difference (MD) with 95% confidence intervals (CIs). We included cluster-randomized trials in the analyses along with individually randomized trials and therefore their sample sizes were adjusted by the methods described in the Cochrane Handbook (43) using a design effect reported from the trial.

We have set out the mortality outcomes of the review in summary of findings tables prepared using the GRADE approach (44) using GRADE profiler software. For each of these outcomes, we assessed the quality of the evidence, considering within-study risk of bias (methodological quality), directness of evidence, heterogeneity, precision of effect estimates, and risk of publication bias. We have rated the quality of the body of evidence for each key outcome as ‘high’, ‘moderate’, ‘low’, or ‘very low’.

The level of attrition was noted for each study. Heterogeneity between trials was assessed using the I-squared statistic, *P* value of <0.1 (χ^2^), and by visual inspection of forest plots. When high levels of heterogeneity between trials (I-squared exceeding 50%) were identified, further exploration was conducted by subgroup analysis and was tested by interaction tests. We applied random-effects meta-analysis as an overall summary when substantial methodological heterogeneity between and among the studies was found. *A priori* subgroup analyses were planned to identify the impact on health care seeking with different strategies (community mobilization, home visitation, combination of two, or perinatal health care/education); and the extent of intervention (birth preparedness, birth preparedness, and recognition and referrals), or (birth preparedness, recognition and referrals and funds for emergency transportation). Potential publication bias was assessed using funnel plots (45).

## Results

**Fig. 1:**
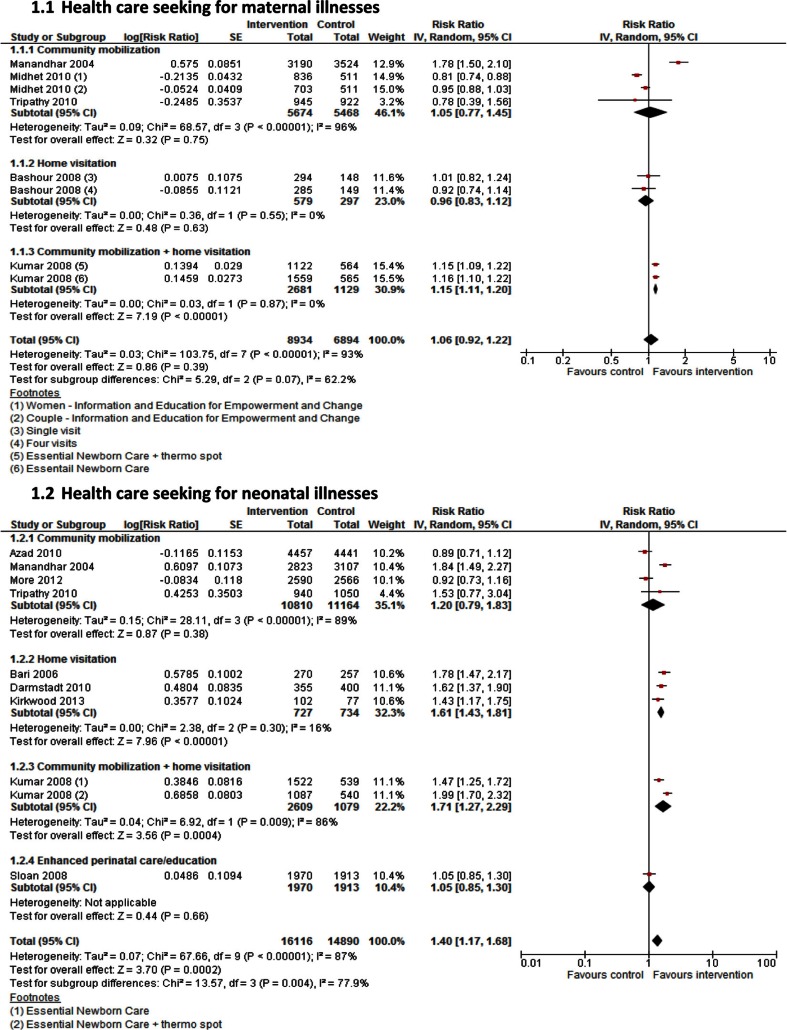
Health care seeking for maternal and ewborn illnesses: intensity of intervention.

Our initial search yielded 20,627 articles, 389 of which had relevant titles and abstracts. After reading the full text of these, 72 appeared to meet our inclusion criteria (Panel 2). After finding 14 of these 72 articles did not meet our inclusion criteria, we included and analyzed 58 original studies (90 published papers), of which 29 were RCTs, 15 were non-RCTs, and 14 were before-after studies (Fig. 1) (characteristics of included studies – Supplementary File 1).

**Panel 2:**
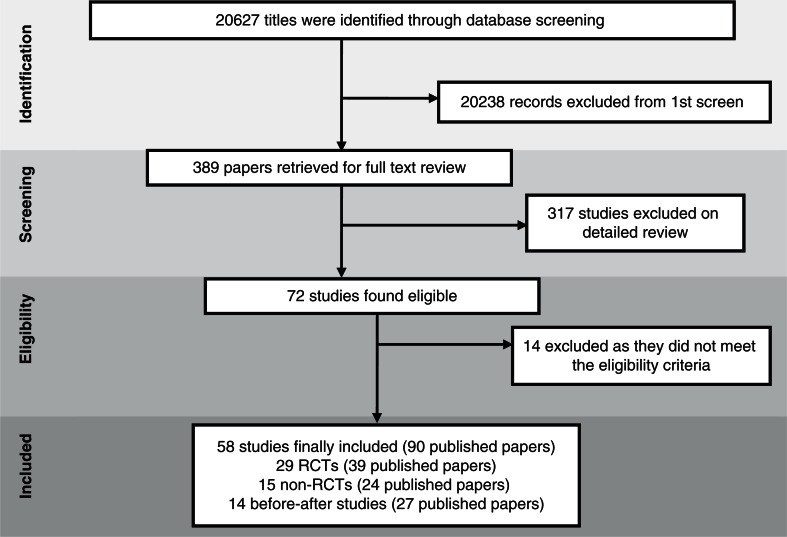
Search flow diagram.

A variety of different interventions and behaviors were assessed in the studies that met the eligibility criteria for inclusion (Panel 3). These interventions and behaviors included promoting routine antenatal care, institutional births, and early breastfeeding; provision of clean delivery kits; training of CHWs, SBA, and health care staff on birth preparedness; and provision of maternal and newborn health interventions. In several included studies these interventions were provided in the form of packages of different strategies including community mobilization, home visitation, or a combination of two.

**Panel 3:** Types of different interventions provided in included studies

Antenatal interventions		Intrapartum interventions		Postnatal interventions		Others	
•	Promotion of routine antenatal care check-ups	•	Provision of safe delivery kits	•	Promotion of early and exclusive breastfeeding	•	TBA/CHW training
•	Tetanus toxoid immunization	•	Clean delivery practices	•	Kangaroo mother care/ thermoregulation	•	Advocacy group meeting
•	Nutritional counseling	•	Referrals for complications and emergencies	•	Newborn resuscitation	•	Counseling and one-to-one session regarding birth preparedness and newborn care
•	Iron/folate supplementation			•	Case management of pneumonia	•	Training staff at health facility
•	Maternal health education			•	Recognition of neonatal danger signs	•	Provision of drugs and supplies at health facilities
•	Promotion of institutional deliveries			•	Referrals for sick newborn		
•	Promotion of clean delivery kits			•	Postnatal visitation		
•	Promotion of breastfeeding						
•	Skin to skin care for newborns						
•	Care for umbilical cord						

### Primary outcomes: maternal and neonatal health care seeking

Meta-analyses of 27 RCTs (Table 1) with a range of different interventions (Panel 2) showed a 40% increase in health care seeking for neonatal illnesses when compared with standard/no care (RR 1.40; 95 CI: 1.17–1.68; 9 studies, *n=*30,572). However, no significant impact was seen in improving health care seeking for maternal illnesses (RR 1.06; 95% CI: 0.92–1.22; 5 studies, *n=*15,828). Heterogeneity was more than 85% for both these primary outcomes (Fig. 1a and b).

**Table 1:** Results from randomized controlled trials

Outcomes	Summary estimates	Number of studies and participants	Heterogeneity
Primary outcomes			
Health care seeking for maternal illnesses	RR 1.06; 95% CI: 0.92, 1.22	5 (*n=*15,828)	τ^2^ 0.03; χ^2^ *P*<0.00001; *I* ^2^ 93%
Health care seeking for neonatal illnesses	RR 1.40; 95% CI: 1.17, 1.68	9 (*n=*31,006)	τ^2^ 0.07; χ^2^ *P*<0.00001; *I* ^2^ 87%
Secondary outcomes			
Mortality outcomes			
Maternal mortality	RR 0.80; 95% CI: 0.65, 1.00	8 (*n=*114,196)	τ^2^ 0.03; χ^2^ *P*=0.07; *I* ^2^ 30%
Neonatal mortality	**RR 0.80; 95% CI: 0.72, 0.89**	21 (*n=*248,848)	τ^2^ 0.06; χ^2^ *P*<0.00001; *I* ^2^ 83%
Early neonatal mortality	**RR 0.70; 95% CI: 0.61, 0.81**	11 (*n=*113,147)	τ^2^ 0.05; χ^2^ *P*<0.00001; *I* ^2^ 77%
Late neonatal mortality	**RR 0.77; 95% CI: 0.64, 0.93**	9 (*n=*108,359)	τ^2^ 0.03; χ^2^ *P*=0.08; *I* ^2^ 42%
Stillbirths	**RR 0.82; 95% CI: 0.74, 0.92**	12 (*n=*176,683)	τ^2^ 0.03; χ^2^ *P*=0.0002; *I* ^2^ 68%
Perinatal mortality	**RR 0.84; 95% CI: 0.78, 0.90**	16 (*n=*279,618)	τ^2^ 0.02; χ^2^ *P*<0.00001; *I* ^2^ 68%
Morbidity outcomes			
Any perceived maternal illnesses	RR 0.87; 95% CI: 0.65, 1.15	3 (*n=*26,005)	τ^2^ 0.00; χ^2^ *P*=0.55; *I* ^2^ 0%
Any perceived neonatal illnesses	**RR 0.61; 95% CI: 0.43, 0.85**	2 (*n=*12,019)	τ^2^ 0.00; χ^2^ *P*=0.79; *I* ^2^ 0%
Process outcomes			
Any antenatal care	**RR 1.26; 95% CI: 1.16, 1.37**	13 (*n=*141,006)	τ^2^ 0.02; χ^2^ *P*<0.00001; *I* ^2^ 96%
Any tetanus toxoid immunization	**RR 1.07; 95% CI: 1.04, 1.11**	8 (*n=*83,243)	τ^2^ 0.00; χ^2^ *P*<0.00001; *I* ^2^ 81%
Iron/folate supplementation	**RR 1.49; 95% CI: 1.06, 2.11**	6 (*n=*81,706)	τ^2^ 0.23; χ^2^ *P*<0.00001; *I* ^2^ 99%
Birthing by skilled birth attendant	RR 1.15; 95% CI: 0.99, 1.34	7 (*n=*53,583)	τ^2^ 0.04; χ^2^ *P*<0.00001; *I* ^2^ 89%
Institutional births	**RR 1.15; 95% CI: 1.05, 1.26**	16 (*n=*116,848)	τ^2^ 0.03; χ^2^ *P*<0.00001; *I* ^2^ 84%
Initiation of breastfeeding within an hour of birth	**RR 1.77; 95% CI: 1.43, 2.19**	14 (*n=*100,272)	τ^2^ 0.16; χ^2^ *P*<0.00001; *I* ^2^ 98%

Significant estimates are provided in BOLD.

Subgroup analyses, based on intensity of interventions, suggested that birth preparedness alone as an intervention had no impact on improving health care seeking for maternal illnesses (RR 1.26; 95% CI: 0.57–2.80; 2 studies, *n=*8,581) or newborn illnesses (RR 1.16; 95% CI: 0.85–1.59; 5 studies, *n=*25,857). Similarly, recognition and referrals for maternal complications showed no impact on improving health care seeking for maternal illnesses (RR 0.96; 95% CI: 0.83–1.12; 1 study, *n=*876). When birth preparedness counseling was combined with recognition of illnesses and provision of referrals by CHWs, health care seeking improved for both maternal illnesses (RR 1.15; 95% CI: 1.11–1.20; 1 study, *n=*3,810) and newborn illnesses (RR 1.65; 95% CI: 1.46–1.86; 4 studies, *n=*4,715). However, when birth preparedness was combined with recognition and referrals along with collecting funds for emergency transportation, it showed no impact on improving maternal health care seeking (RR 0.88; 95% CI: 0.75–1.03, 1 study, *n=*2,561) (Fig. 1a and b).

**Fig. 2:**
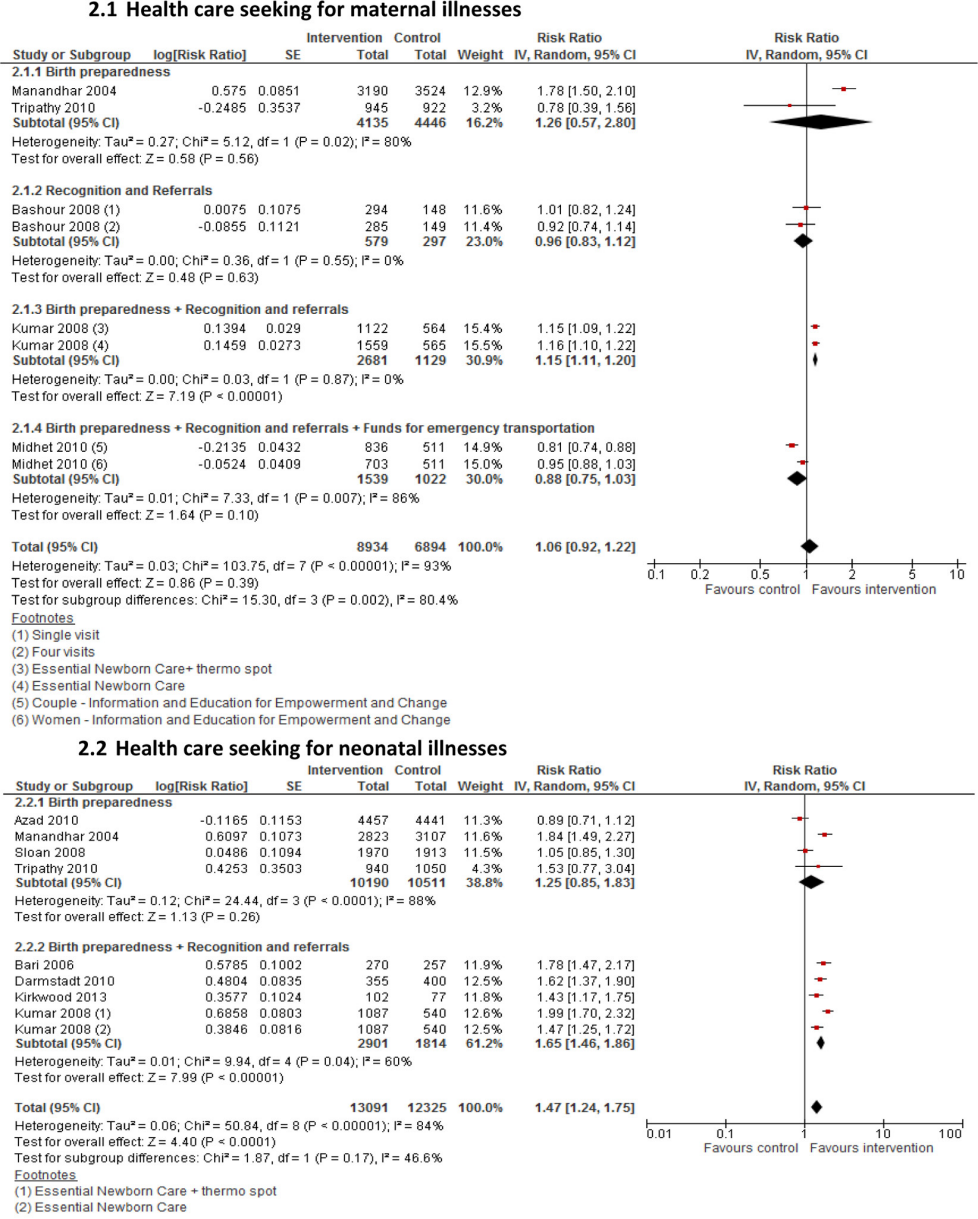
Health care seeking for maternal and newborn illnesses: strategies for delivering interventions.

Based on strategies used for enhancing health care seeking, community mobilization alone showed no improvement in health care seeking for maternal illnesses (RR 1.05; 95% CI: 0.77–1.45, 3 studies, *n=*11,144) or neonatal illnesses (RR 1.20; 95% CI: 0.79–1.83, 4 studies, *n=*21,974,). Home visiting by CHWs alone had a significant impact on improving health care seeking for neonatal illnesses (RR 1.61; 95% CI: 1.43–1.81; 3 studies, *n=*1,461), but no impact was seen for maternal illnesses (RR 0.96; 95% CI: 0.83–1.12, 1 study, *n=*876). When home visiting was combined with community mobilization, significant improvements were seen for both maternal illnesses (RR 1.15; 95% CI: 1.11–1.20; 1 study, *n=*3,810) and newborn illnesses (RR 1.71; 95% CI: 1.27–2.29; 1 study, *n=*3,688) (Fig. 2a and b). Estimates from non-RCTs found no impact on improving health care seeking for neonatal illnesses (Table 2). However, results from before-after studies were similar to the results from RCTs showing no impact on health care seeking for maternal illnesses RR 1.13; 95% CI: 0.86–1.48; 1 study, *n=*1,443 but significant improvement in health care seeking for neonatal illnesses (RR 1.35; 95% CI: 1.19–1.53; 4 studies, *n=*4,348) (Table 3).

**Table 2:** Results from non-randomized controlled trials

Outcomes	Summary estimates	Number of studies and participants	Heterogeneity
Primary outcomes			
Health care seeking for neonatal illnesses	RR 0.96; 95% CI: 0.71, 1.31	3 (*n=* 2,103)	τ^2^ 0.09; χ^2^ *P*<0.00001; *I* ^2^ 94%
Secondary outcomes			
Mortality outcomes			
Maternal mortality	RR 0.97; 95% CI: 0.64, 1.49	5 (*n=*119,078)	τ^2^ 0.19; χ^2^ *P*<0.00001; *I* ^2^ 89%
Neonatal mortality	RR 0.83; 95% CI: 0.54, 1.26	4 (*n=*28,641)	τ^2^ 0.13; χ^2^ *P*=0.004; *I* ^2^ 77%
Early neonatal mortality	RR 0.57; 95% CI: 0.30, 1.09	2 (*n=*3,921)	τ^2^ 0.10; χ^2^ *P*=0.19; *I* ^2^ 41%
Late neonatal mortality	RR 0.84; 95% CI: 0.12, 5.80	2 (*n=*3,921)	τ^2^ 1.55; χ^2^ *P*=0.03; *I* ^2^ 79%
Stillbirths	RR 0.97; 95% CI: 0.71, 1.34	3 (*n=*6,096)	χ^2^ *P*=0.01; *I* ^2^ 77%
Perinatal mortality	RR 0.74; 95% CI: 0.44, 1.23	4 (*n=*101,834)	τ^2^ 0.22; χ^2^ *P*<0.00001; *I* ^2^ 89%
Morbidity outcomes			
Any perceived neonatal illnesses	RR 1.12; 95% CI: 0.90, 1.39	1 (*n=*459)	
Process outcomes			
Any antenatal care	**RR 1.05; 95% CI: 1.04, 1.06**	3 (*n=*31,305)	χ^2^ *P*< 0.00001; *I* ^2^ 98%
Iron/folate supplementation	**RR 24.53; 95% CI: 13.20, 45.59**	1 (*n=*756)	–
Birthing by skilled birth attendant	RR 1.03; 95% CI: 0.97, 1.10	1 (*n=*13,826)	–
Institutional births	**RR 1.89; 95% CI: 1.48, 2.41**	2 (*n=*2,291)	τ^2^ 0.03; χ^2^ *P*<0.00001; *I* ^2^ 86%
Initiation of breastfeeding within an hour of birth	**RR 6.54; 95% CI: 5.88, 7.27**	1 (*n*=13,826)	–

Significant estimates are provided in BOLD.

**Table 3:** Results from before/after studies

Outcomes	Summary estimates	Number of studies and participants	Heterogeneity
Primary outcomes			
Health care seeking for maternal illnesses	RR 1.13; 95% CI: 0.86, 1.48	1 (*n=* 1,443)	–
Health care seeking for neonatal illnesses	**RR 1.35; 95% CI: 1.19, 1.53**	4 (*n=* 4,348)	τ^2^ 0.01; χ^2^ *P*=0.003; *I*^2^ 75%
Secondary outcomes			
Mortality outcomes			
Neonatal mortality	RR 0.55; 95% CI: 0.18, 1.73	2 (*n=* 60,762)	τ^2^ 0.66; χ^2^ *P*< 0.00001; *I*^2^ 98%
Early neonatal mortality	RR 1.53; 95% CI: 0.78, 3.01	3 (*n=* 3,418)	τ^2^ *P* 0.26; χ^2^ *P*=0.004; *I*^2^ 82%
Stillbirths	**RR 0.70; 95% CI: 0.60, 0.82**	4 (*n=* 61,176)	χ^2^ *P* 0.03; *I*^2^ 65%
Perinatal mortality	RR 0.96; 95% CI: 0.85, 1.09	4 (*n=* 60,944)	χ^2^ *P*< 0.00001; *I*^2^ 90%
Process outcomes			
Any antenatal care	**RR 1.27; 95% CI: 1.24, 1.30**	3 (*n=* 10,137)	χ^2^ *P*< 0.00001; *I*^2^ 98%
Iron/folate supplementation	**RR 1.29; 95% CI: 1.25, 1.33**	1 (*n=* 3,480)	–
Any tetanus toxoid immunization	**RR 1.14; 95% CI: 1.10, 1.17**	1 (*n=* 3,480)	
Institutional births	RR 32.76; 95% CI: 0.04, 29028.97	2 (*n=* 5,859)	τ^2^ = 23.02; χ^2^ *P*< 0.00001; *I*^2^ 96%
Initiation of breastfeeding within an hour of birth	RR 1.54; 95% CI: 0.97, 2.44	2 (*n* = 2,474)	τ^2^ = 0.11; χ^2^ *P*< 0.00001; *I*^2^ 99%

Significant estimates are provided in BOLD.

### Mortality outcomes

**Fig. 3:**
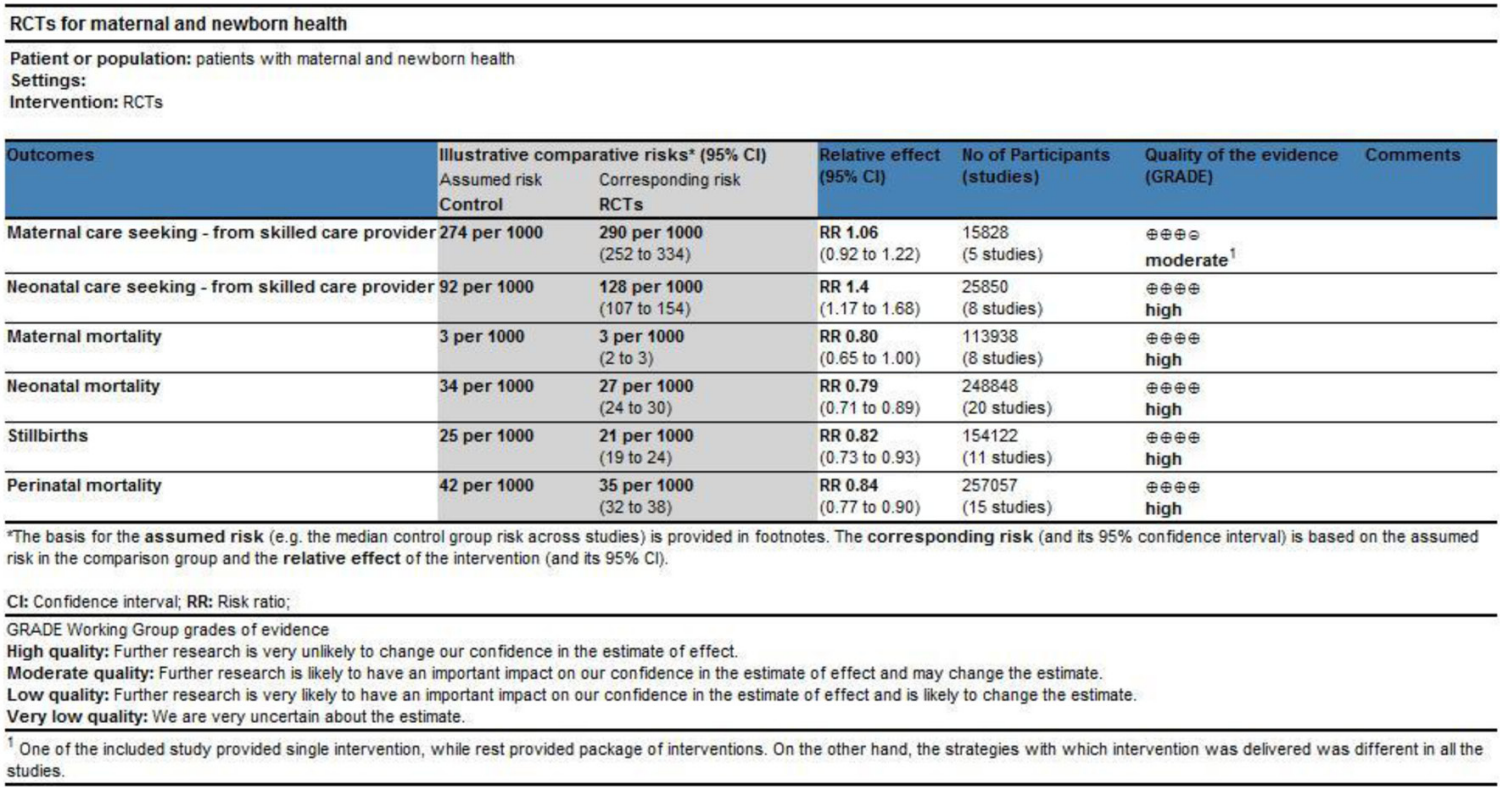
GRADE analysis.

RCTs included in this review displayed a non-significant borderline reduction in maternal mortality (RR 0.80; 95% CI: 0.65–1.00; 8 studies, *n=*114,196) (Table 1). However, significant impact was observed on total neonatal mortality (RR 0.80; 95% CI: 0.72–0.89; 21 studies, *n=*248,848) (Supplementary File 2) including both early (RR 0.70; 95% CI: 0.61–0.81; 11 studies, *n=*113,147) and late neonatal mortality (RR 0.77; 95% CI: 0.6–0.93; 9 studies, *n=*108,359) (Table 1). Impact was also significant for reducing perinatal mortality (RR 0.84; 95% CI: 0.78–0.90; 16 studies, *n=*279,618) and stillbirths (RR 0.82; 95% CI: 0.74–0.92; 12 studies, *n=*176,683) (Table 1). On GRADE analysis, evidence was moderate for maternal mortality; however, it was high quality for the rest of the other mortality outcomes (Fig. 3).

Based on intensity of interventions and strategies used within RCTs, no differences were seen for reducing maternal mortality (Table 4). Community mobilization alone showed a significant impact on reducing neonatal mortality (RR 0.79; 95% CI: 0.70–0.89; 10 studies, *n=*123,047); however, home visitation alone showed no impact (RR 0.87; 95% CI: 0.57–1.32; 4 studies, *n=*21,214). Community mobilization and home visitation in combination showed a significant impact on reducing neonatal mortality (RR 0.73; 95% CI: 0.71–0.89; 4 studies, *n=*114,509), and enhanced perinatal care/education showed no impact (RR 0.90; 95% CI: 0.57–1.41; 4 studies, *n=*12,455). Community mobilization alone (RR 0.69; 95% CI: 0.57–0.82; 6 studies, *n=*73,288) and community mobilization in combination with home visitation (RR 0.69; 95% CI: 0.54–0.88; 3 studies, *n=*32,263) showed a significant impact on reducing early neonatal mortality (ENM); however, enhanced perinatal care/education alone had no impact (RR 0.81; 95% CI: 0.44–1.50; 2 studies, *n=*7,569). Community mobilization in combination with home visitation also showed significant impact on reducing late neonatal mortality (RR 0.61; 95% CI: 0.41–0.92; 3 studies, *n=*32,263), however, community mobilization (RR 0.83; 95% CI: 0.67–1.03; 5 studies, *n=*71,931) together with enhanced perinatal care/education showed no impact (RR 1.09; 95% CI: 0.55–2.18; 1 study, *n=*4,165). Community mobilization in combination with home visitation (RR 0.78; 95% CI: 0.71–0.86; 2 studies, *n=*33,786) and enhanced perinatal care/education (RR 0.62; 95% CI: 0.49–0.79; 2 studies, *n=*6,251) showed a significant impact on reducing stillbirths, however, community mobilization alone showed no impact (RR 0.94; 95% CI: 0.83–1.06; 7 studies, *n=*136,646). Community mobilization in combination with home visitation (RR 0.78; 95% CI: 0.68–0.89; 5 studies, *n=*100,553) and community mobilization alone (RR 0.88; 95% CI: 0.82–0.95; 10 studies, *n=*205,843) showed a significant impact on reducing stillbirths, however, enhanced perinatal care/education alone showed no impact (RR 0.84; 95% CI: 0.61–1.16; 3 studies, *n=*27,326) (Table 4).

**Table 4:** Mortality outcomes based on intensity of interventions and strategies employed

Strategies employed					
	Community mobilization	Home visitation	Community mobilization and home visitation	Enhanced perinatal care/education	Subgroup differences (*P* value of Chi^2^)
Maternal mortality	RR 0.81 95% CI: 0.54, 1.21 6 studies, *n=*79,203 τ^2^ 0.13; χ^2^ *P*=0.04; *I*^2^ 56%	RR 0.62; 95% CI: 0.35, 1.09 2 studies, *n=*10,021 τ^2^ 0.00; χ^2^ *P*=0.50; *I*^2^ 0%	RR 0.82; 95% CI: 0.46, 1.46 1 study, *n=*6,230	RR 0.74; 95% CI: 0.45, 1.22 1 study, *n=*18,699	*P*=0.16
Neonatal mortality	**RR 0.79; 95% CI: 0.70, 0.89** **10 studies**,***n=*****123,047** **τ^2^ 0.03;** χ**^2^*****P***=**0.0001;*****I*****^2^ 71%**	RR 0.87; 95% CI: 0.57, 1.32 4 studies, *n=*21,214 τ^2^ 0.12; χ^2^ *P*=0.01; *I*^2^ 70%	**RR 0.73; 95% CI: 0.71, 0.89** **4 studies**, ***n=*****114,509** **τ^2^ 0.05;** χ**^2^*****P***=**0.74;*****I*****^2^ 83%**	RR 0.90; 95% CI: 0.57, 1.41 4 studies, *n=*12,455 τ^2^ 0.19; χ^2^ *P*<0.0001; *I*^2^ 94%	*P*=0.78
Early neonatal mortality	**RR 0.69; 95% CI: 0.57, 0.82** **6 studies**, ***n=*** **73,288** **τ^2^ 0.05;** χ**^2^** ***P*** **<0.00001;** ***I*** **^2^ 81%**	–	**RR 0.69; 95% CI: 0.54, 0.88** **3 studies**, ***n=*** **32,263** **τ^2^ 0.04;** χ**^2^** ***P***=**0.02;** ***I*** **^2^ 68%**	RR 0.81; 95% CI: 0.44, 1.50 2 studies, *n=*7,596 τ^2^ 0.14; χ^2^ *P*=0.01; *I* ^2^ 70%	**P <0.0001**
Late neonatal mortality	RR 0.83; 95% CI: 0.67, 1.03 5 studies, *n=*71,931 τ^2^ 0.02; χ^2^ *P*=0.22; *I* ^2^ 31%	–	**RR 0.61; 95% CI: 0.41, 0.92** **3 studies**, ***n=*** **32,263** **τ^2^ 0.10;** χ**^2^** ***P***=**0.04;** ***I*** **^2^ 63%**	RR 1.09; 95% CI: 0.55, 2.18 1 study, n4,165	
	*P*= 0.28				
Stillbirths	RR 0.94; 95% CI: 0.83, 1.06 7 studies, *n=*136,646 τ^2^ 0.01; χ^2^ *P*=0.09; *I* ^2^ 45%	**–**	**RR 0.78; 95% CI: 0.71, 0.86** **2 studies**, ***n=*** **33,786** **τ^2^ 0.00;** χ**^2^** ***P***=**0.74;** ***I*** **^2^ 0%**	**RR 0.62; 95% CI: 0.49, 0.79** **2 studies**, ***n=*** **6,251** τ**^2^ 0.01;** χ**^2^** ***P***=**0.16;** ***I*** **^2^ 50%**	**P**=**0.004**
Perinatal mortality	**RR 0.88; 95% CI: 0.82, 0.95** **10 studies**, ***n=*** **205,843** **τ^2^ 0.01;** χ**^2^** ***P***=**0.08;** ***I*** **^2^ 41%**	RR 0.82; 95% CI: 0.62, 1.08 1 study, *n=*6,376	**RR 0.78; 95% CI: 0.68, 0.89** **5 studies**, ***n=*** **100,553** **τ^2^ 0.02;** χ**^2^** ***P***=**0.002;** ***I*** **^2^ 73%**	RR 0.84; 95% CI: 0.61, 1.16 3 studies, *n=*27,326 τ^2^ 0.07; χ^2^ *P*=0.005; *I* ^2^ 88%	*P*=0.41

**Intensity of intervention**					

	Birth preparedness	Birth preparedness + recognition and referrals	Birth preparedness + recognition and referrals + Funds for emergency transportation		

Maternal mortality	RR 0.81 95% CI: 0.54, 1.21 6 studies, *n=*80,040 τ^2^ 0.13; χ^2^ *P*=0.04; *I* ^2^ 56%	RR 0.73; 95% CI: 0.51, 1.05 3 studies, *n=*29,454 τ^2^ 0.00; χ^2^ *P*=0.63; *I* ^2^ 0%	–		*P*=0.71
Neonatal mortality	RR 0.91; 95% CI: 0.76, 1.09 11 studies, *n=*129,937 τ^2^ 0.08; χ^2^ *P*<0.00001; *I* ^2^ 85%	RR 0.73; 95% CI: 0.60, 0.88* **8 studies**, ***n=*** **105,846** **τ^2^ 0.07;** χ**^2^** ***P*** **<0.00001;** ***I*** **^2^ 79%**	**RR 0.78; 95% CI: 0.70, 0.87** **2 studies**, ***n=*** **29,927** **τ^2^ 0.03;** χ**^2^** ***P***=**0.0001;** ***I*** **^2^ 0%**		*P*=0.11
Early neonatal mortality	**RR 0.79; 95% CI: 0.66, 0.95** **6 studies**, ***n=*** **75,196** **τ^2^ 0.03;** χ**^2^** ***P***=**0.010;** ***I*** **^2^ 67%**	**RR 0.55; 95% CI: 0.43, 0.71** **2 studies**, ***n=*** **7,119** **τ^2^ 0.00;** χ**^2^** ***P***=**0.98;** ***I*** **^2^ 0%**	**RR 0.66; 95% CI: 0.50, 0.88** **3 studies**, ***n=*** **30,832** **τ^2^ 0.08;** χ**^2^** ***P*** **<0.0001;** ***I*** **^2^ 90%**		***P***=**0.06**
Late neonatal mortality	RR 0.85; 95% CI: 0.70, 1.04 6 studies, *n=*76,096 τ^2^ 0.01; χ^2^ *P*=0.28; *I* ^2^ 21%	**RR 0.40; 95% CI: 0.24, 0.68** **1 study**, ***n=*** **3,688** **τ^2^ 0.00;** χ**^2^** ***P***=**0.43;** ***I*** **^2^ 0%**	RR 0.80; 95% CI: 0.60, 1.06 2 studies, *n=*28,575 τ^2^ 0.01; χ^2^ *P*=0.23; *I* ^2^ 30%		***P***=**0.03**
Stillbirths	**RR 0.85; 95% CI: 0.74, 0.96** **10 studies, 171,703** τ**^2^ 0.03;** χ**^2^** ***P***=**0.0002;** ***I*** **^2^ 72%**	**RR 0.72; 95% CI: 0.61, 0.84** **3 studies**, ***n=*** **4,980** **τ^2^ 0.00;** χ**^2^** ***P***=**0.067;** ***I*** **^2^ 0%**	**RR 0.78; 95% CI: 0.70, 0.87** **2 studies**, ***n=*** **29,927** **τ^2^ 0.03;** χ**^2^** ***P***=**0.0001;** ***I*** **^2^ 0%**		*P*=0.28
Perinatal mortality	**RR 0.91; 95% CI: 0.84, 0.98** **9 studies**, ***n=*** **149,079** τ**^2^ 0.01;** χ**^2^** ***P***=**0.05;** ***I*** **^2^ 48%**	**RR 0.75; 95% CI: 0.64, 0.89** **5 studies**, ***n=*** **93,665** **τ^2^ 0.03;** χ**^2^** ***P***=**0.003;** ***I*** **^2^ 72%**	RR 0.81; 95% CI: 0.64, 1.01 3 studies, *n=*32,184 τ^2^ 0.03; χ^2^ *P*=0.008; *I* ^2^ 75%		*P*=0.11

Only Bashour 2008 (with 2 subgroups – single visit and 4 visits) did not have birth preparedness component in the intervention. Significant estimates are provided in BOLD.

Subgroup analyses based on intensity of interventions showed birth preparedness alone had no impact on improving neonatal mortality (RR 0.91; 95% CI: 0.76–1.09; 11 studies, *n=*129,937), however, when birth preparedness was paired with recognition and referrals (RR 0.73; 95% CI: 0.60–0.88; 8 studies, *n=*105,846) and then with funds for emergency transportation (RR 0.78; 95% CI: 0.70–0.87; 2 studies, *n=*29,927) there was a significant impact on reducing neonatal mortality. Similarly, birth preparedness alone showed no impact on improving late neonatal mortality (RR 0.85; 95% CI: 0.70–1.04; 6 studies, *n=*76,096), however, when birth preparedness was paired with recognition and referrals (RR 0.40; 95% CI: 0.24–0.68; 1 study, *n=*3,688) there was a significant impact on reducing late neonatal mortality. When funds for emergency transportation was combined with birth preparedness and recognition and referrals (RR 0.80; 95% CI: 0.70–0.87; 2 studies, *n=*29,927). Birth preparedness alone showed a significant impact on reducing ENM and stillbirths (ENM RR 0.79; 95% CI: 0.66–0.95; 6 studies, *n=*75,196; stillbirths RR 0.85; 95% CI: 0.74–0.96; 10 studies, *n=*171,703); it also showed an impact on these outcomes when paired with recognition of complication and referrals (ENM RR 0.55; 95% CI: 0.43–0.71; 2 studies, *n=*7,119; stillbirths RR 0.72; 95% CI: 0.61–0.84; 3 studies, *n=*4,980) and funds for emergency transportation (ENM RR 0.66; 95% CI: 0.50–0.88; 3 studies, *n=*30,832; stillbirths RR 0.78; 95% CI: 0.70–0.87; 2 studies, *n=*29,927). Birth preparedness alone (RR 0.91; 95% CI: 0.84–0.98; 9 studies, *n=*149,097) and in combination to recognition and referrals (RR 0.75; 95% CI: 0.64–0.89; 5 studies, *n=*93,665) showed significant impact on reducing perinatal mortality, however when further intensified and paired with funds for emergency transportation (RR 0.81; 95% CI: 0.64–1.01; 3 studies, *n=*32,184), there was no impact on reducing perinatal mortality (Table 4).

From non-RCTs (Table 2) and before-after studies (Table 3), significant reductions in stillbirths were observed (RR 0.70; 95% CI: 0.60–0.82; 4 studies, *n=*61,176) (Table 3).

### Morbidity outcomes

From RCTs, significant results were observed in reducing any perceived illnesses in newborns (RR 0.61; 95% CI: 0.43–0.85; 2 studies, *n=*12,019), however, no improvements were observed for maternal illnesses (RR 0.87; 95% CI: 0.65–1.15; 3 studies, *n=*26,005) (Table 1). Results from non-RCTs were not significant for neonatal illnesses (RR 1.12; 95% CI: 0.90–1.39; 1 study, *n=*459) (Table 2).

### Other care seeking outcomes

The review identified a number of RCTs which, when pooled, displayed a positive impact on care practices; these include antenatal care (RR 1.26; 95% CI: 1.16–1.37; 13 studies, *n=*14,1006), receiving tetanus toxoid immunization (RR 1.07; 95% CI: 1.04–1.11; 8 studies, *n=*83,243), and iron/folate supplementation (RR 1.49; 95% CI: 1.06–2.11; 6 studies, *n=*81,706) (Table 1). Improved rates of institutional births (RR 1.15; 95% CI: 1.05–1.26; 16 studies, *n=*116,848) and initiating breastfeeding within an hour of birth (RR 1.77; 95% CI: 1.43–2.19; 14 studies, *n=*100,272) were also seen. However, no improvements were seen for SBA (RR 1.15; 95% CI: 0.99–1.34; 7 studies, *n=*53,583).

For non-RCTS, similar significant improvements were observed on uptake of antenatal care (RR 1.05; 95% CI: 1.04–1.06; 3 studies, *n=*31,305), iron/folate supplementation (RR 24.53; 95% CI: 13.20–45.59; 1 study, *n=*756), institutional births (RR 1.89; 95% CI: 1.48–2.41; 2 studies, *n=*2,291), and initiation of breastfeeding within an hour of birth (RR 6.54; 95% CI: 5.88–7.27; 1 study, *n=*13,826). However, no improvements were observed for women birthing with a SBA (RR 1.03; 95% CI: 0.97–1.10; 1 study, *n=*13,826) (Table 2).

For before-after studies, significant impact was observed for uptake of any antenatal care (RR 1.27; 95% CI: 1.24–1.30; 3 studies, *n=*10137), tetanus toxoid immunization (RR 1.14; 95% CI: 1.10–1.17; 1 study, *n=*3,480), and iron/folate supplementation (RR 1.29; 95% CI: 1.25–1.33; 1 study, *n=*3,480) after an intervention was delivered but not for institutional births or early initiation of breastfeeding (Table 3).

## Discussion

Adequately addressing women's and children's health care needs would resolve a considerable proportion of global health problems. Improving health care seeking for the health of mothers and newborns can prevent many avoidable deaths. Although there was a paucity of included studies reporting health care seeking as an outcome, the systematic review found promising results of the several interventions for improving health care seeking for maternal and newborn illnesses. Although the impact was not significant for health care seeking for maternal illnesses, care seeking for neonatal illnesses improved by 40% overall. The impact was enhanced when the intervention was provided by CHWs though home visiting (45% increase) or when combined with community mobilization (62%), however the later evidence came from a single study with a positive impact. Impact was even larger when promotion of birth preparedness was combined with interventions where CHW recognized illnesses and provided referrals (65% increase). While interpreting the results, it is important to consider that studies were not similar across the subgroups for health care seeking for maternal illnesses and neonatal illnesses.

The included studies did not find any impact for any of these interventions on improving maternal mortality. Probably these studies were not powered to detect small but important differences. Significant improvements were observed for neonatal mortality (21% reduction) including early (30%) and late neonatal mortality (23%), stillbirths (18%), and perinatal mortality (18%). A similar direction of effect, although not significant, was found from non-RCTs and before/after studies. Although impact on mortality was more convincing when interventions were given in the form of community mobilization in combination with home visiting, the degree of heterogeneity was high. Mortality substantially improved when birth preparedness was combined with recognition of illnesses and provision of referrals; and was even more effective when interventions involved collection of funds for emergency transportation. However, the number of studies with increasing intensity of intervention decreased and there were too few studies in the highest level of intensity to make robust claims.

The review found positive impacts for these interventions from RCTs on improving antenatal care (27%), uptake of tetanus toxoid immunization (8%), iron/folate supplementation (49%), institutional births (16%), and initiation of breastfeeding (85%). Similar direction of effects was observed from other less rigorous study designs.

The subgroup analyses suggest that the impacts on health care seeking, mortality, and morbidity were greater when interventions included recognition of illnesses and provision of referrals. However, the qualitative findings from these trials were scarce and little or no information was provided to relate these findings with the contextual factors of delays in those scenarios. The literature suggests health service demand is not determined by recognition of problems and perceived seriousness alone; there are underlying beliefs which play a vital role in determining health care utilization patterns (46–48). Ineffective or inequitable health decision making at the household level is a major obstacles in accessing health care (49, 50). Timely recognition of danger signs, autonomy of decision making, availability of finances, accessibility of the health facility, and perceived quality of care are necessary considerations when making the decision to seek formal care.

Even though modest improvement in maternal and neonatal health outcomes has been achieved in the last decade, these can be further improved. While the use of advocacy groups and mobilization campaigns can help to optimize the implementation of these strategies; health system investment training the community and facility health staff and equipping them with essential supplies can help them care for a high risk pregnancy, as well as respond to any emergency that may arise. A specific implementation strategy could be the provision of birthing kits to the Traditional Birth Attendant (TBA)s which will ensure access for those residing in remote areas. This is likely to reduce mortality arising from delay in the provision of emergency medical aid during childbirth. In addition, indirect health care costs such as transportation and certain minor charges at the facility should be minimized. Full implementation of these changes will go a long way to improve not only maternal and neonatal health-seeking behavior, but also their health outcomes.

## Conclusions

This systematic review identified that strategies such as mobilization and home visitation can improve health care seeking for neonatal illnesses and can reduce perinatal mortality. Further analyses based on strategies which combined birth preparedness counseling with recognition of illnesses and provision of referrals by CHWs showed an improvement in both maternal and neonatal health care seeking. Similarly, strategies which used mobilization with home visitation showed an improvement in both maternal and newborn health care seeking; however the evidence was only derived from a single study. These interventions had a significant impact on reducing stillbirths, perinatal deaths, and neonatal mortality. Most of the included studies were conducted in Asia, with very a limited number of studies from other LMIC countries such as Africa. Thus, there is a clear need for additional high quality research from other LMIC regions. There is also a need to identify the cost-effectiveness of identified strategies to provide interventions in affordable ways to hard-to-reach communities to prevent illnesses and promote health.

## Supplementary Material

Strategies for improving health care seeking for maternal and newborn illnesses in low- and middle-income countries: a systematic review and meta-analysis

## Paper context

Reducing maternal and newborn mortality will require rigorous efforts to scale up evidence-based interventions, especially community-based preventive, promotive and therapeutic strategies, as well as availability of commodities and health workers in primary care. A lack of appropriate care seeking for ill mothers and neonates is thought to contribute to high mortality rates; therefore, a major challenge is the appropriate mix of strategies for demand creation as well as provision of services. Prior published reviews have systematically assessed the prevalence of care seeking in neonates; however the prevalence of care seeking among mothers has not been systematically assessed. Also there are no reviews which have assessed the strategies or interventions to increase care seeking for maternal and neonatal health. There is a need to systematically review the literature to determine the interventions that can improve the care seeking pattern among mothers and their newborns at primary care setting in developing countries.
