# Supplementary material for: Strategies for improving health care seeking for maternal and newborn illnesses in low- and middle-income countries: a systematic review and meta-analysis
Source: Glob Health Action. 2016 May 10;9:10.3402/gha.v9.31408. doi: 10.3402/gha.v9.31408 (PMC4864851; doi:10.3402/gha.v9.31408)
Supplement: Strategies for improving health care seeking for maternal and newborn illnesses in low- and middle-income countries: a systematic review and meta-analysis [file GHA-9-31408-s001.pdf]

Strategies for improving health care seeking for maternal and newborn illnesses in low and middle income countries: a systematic review and meta-analysis

Zohra S Lassi, Philippa F Middleton, Zulfiqar A Bhutta, Caroline Crowther

Web annex 1:
Characteristics of included studies: RCTs

Author/year	Country	Target population	Intervention	Comparison	Duration of intervention	Outcomes reported	Assessment of bias
Acharya 2015 [1]	India	Expectant mothers (n=11885)	**Community mobilization** To raise awareness of essential maternal and newborn health care through communication and advocacy activities to promote safe pregnancy and neonatal care, directly at the village level and through mass campaigns at a district level. (L1 and L2 areas where they received more intense mobilization that included L1 and strengthening village health and sanitation committees (established at the panchayat level to improve accountability related to supply of funds, facilities, commodities, and services needed to support core maternal and newborn health activities).	No comparison	2 years	Neonatal deaths and other care related outcomes	Selection=LR<br>Performance and detection = LR<br>Attrition=UC<br>Reporting = LR
Fotrell 2013 [2]<br><br>**ISRCTN01805825**	Bangladesh	Women of reproductive age (19301 live births)	**Community mobilization** Participatory learning and action cycle (n=9106)	Standard care (n=10204)	2.5 years	Stillbirths, neonatal deaths, perinatal deaths, maternal deaths, and other care related outcomes	Selection=LR<br>Performance and detection = LR<br>Attrition=LR<br>Reporting = LR
Persson 2013 [3]<br>**ISRCTN44599712**	Vietnam	Women of reproductive age (22561 births)	**Community mobilization** Laywomen facilitated monthly meetings during the 3 years in groups composed of health care staff and key persons in the communes. A problem-solving approach was employed including identifying and prioritizing local perinatal health problems and accomplishing improvement cycles that included concrete actions on prioritization problems (n=11906).	Standard care (n=10655)	3 years	Stillbirths, ,neonatal deaths, maternal deaths, perinatal deaths, other care related outcomes	Selection = LR<br>Performance and detection = LR<br>Attrition = UC<br>Reporting = LR

Author/year	Country	Target population	Intervention	Comparison	Duration of intervention	Outcomes reported	Assessment of bias
Lewycka 2013 [4, 5]<br><br>**ISRCTN06477126**	Malawi	Women of reproductive age (26262 births)	**Community mobilization**<br>Community action cycle and home visits at five time points during pregnancy and after birth (n=13683)<br>**Home visitation**<br>Peer counsellors made five home visits during and after pregnancy and provided education on exclusive breastfeeding, infant care, immunization, MTCT, and family planning (n=14022)<br>**Community mobilization and home visitation**<br>(n=13678)	Standard care (n=14548)	4 years	Stillbirths, neonatal deaths, perinatal deaths, Infant deaths, maternal deaths, and other care related outcomes	Selection=LR<br>Performance and detection = LR<br>Attrition=LR<br>Reporting = LR
Kirkwood 2013 [6]<br><br>**NCT00623337**	Ghana	Pregnant women (16329 births)	**Home visitation**<br>Home visits during pregnancy to promote essential newborn-care practices, assess babies for danger signs, and refer as necessary (n=9885)	Standard care (n=10096)	1 year	Neonatal health care seeking, stillbirths neonatal deaths, perinatal deaths, infant deaths, maternal deaths, and other care related outcomes	Selection=LR<br>Performance and detection = LR<br>Attrition=LR<br>Reporting = LR
Nsibande 2013 [7-9]<br><br>**ISRCTN41046462**	South Africa	Pregnant women (n=2423)	**Home visitation**<br>Home visits made to mothers on uptake of PMTCT interventions and appropriate newborn care practices. Babies with illness or identified danger signs were referred and also assessed the effectiveness of this referral system (n=2423)	Standard care	3 years	Care related outcomes	Selection=UC<br>Performance and detection = LR<br>Attrition=UR<br>Reporting = LR
Magoma 2013 [10]<br><br>**ACTRN 12609000268246**	Tanzania	Pregnant women (905)	**Enhanced Perinatal care/education**<br>Introduction and promotion of birth plans by care providers (n=404)	Standard care (501)	8 months	Perception of quality care, EmONC and postnatal care related outcomes	Selection=LR<br>Performance and detection = HR<br>Attrition=LR<br>Reporting = LR
More 2012 [11, 12]<br><br>**ISRCTN96256793**	India	Women of reproductive age (18197 births)	**Community mobilization**<br>Action learning cycle in which they discussed perinatal experiences, improved their knowledge, and took local action (n=9155 births)	Standard care (n=9042 births)	3 years	Stillbirths, neonatal deaths, extended perinatal deaths, and other care related outcomes	Selection=LR<br>Performance and detection = LR<br>Attrition=LR<br>Reporting = LR
Colbourn 2012 [13]<br><br>**ISRCTN18073903**	Malawi	Women of reproductive age (160576 births)	**Community mobilization**<br>Participatory women's group community intervention (5080 births)	Standard care (4919 births)	2 years	Stillbirths, neonatal deaths, perinatal deaths, maternal deaths, and other care related outcomes	Selection=LR<br>Performance and detection = LR<br>Attrition=LR<br>Reporting = LR

Author/year	Country	Target population	Intervention	Comparison	Duration of intervention	Outcomes reported	Assessment of bias
Bhandari 2012 [14]<br><br>**NCT00474981**	India	29,667 births in intervention clusters and 30,813 in control clusters.	**Community mobilization and home visitation**<br>Community health workers were trained to conduct postnatal home visits and women's group meetings; physicians, nurses, and CHWs were trained to treat or refer sick newborns and children; supply of drugs and supervision were strengthened	Standard care	1.5 years	Stillbirths, neonatal deaths, perinatal deaths, infant deaths, post neonatal deaths, and other care related outcomes	Selection=LR<br>Performance and detection = LR<br>Attrition=UR<br>Reporting = LR
Bhutta 2011 [15, 16]<br><br>**ISRCTN16247511**	Pakistan	Women of reproductive age (26987 pregnancies)	**Community mobilization and home visitation**<br>Creation of volunteer-based village health committees, basic training and linkage of TBAs with LHWs, promotion of antenatal care, immediate newborn care, training in group counselling and communication strategies, recognition of sick newborn babies and danger signs for referral (n=14152)	Standard care (n=12835)	2 years	Stillbirths, neonatal deaths, perinatal deaths, maternal deaths, and other care related outcomes	Selection=LR<br>Performance and detection = LR<br>Attrition=LR<br>Reporting = LR
Wu 2011 [17]<br><br>**NCT 01054235**	China		**Enhanced Perinatal care/education**<br>Training township hospital midwives and instructing them in how to provide systematic maternal care, 2) informing women in the community of the importance of prenatal care, 3) if needed, providing basic medical instruments to the hospitals (n=673)	Standard care (n=591)	2.5 years	Stillbirths, neonatal deaths, perinatal deaths, and other care related outcomes	Selection=LR<br>Performance and detection = LR<br>Attrition=UR<br>Reporting = LR
Gill 2011[18]<br><br>**NCT00518856**	Zambia	3559 infants	**Enhanced Perinatal care/education**<br>Training in a modified version of the neonatal resuscitation protocol, and single dose amoxicillin coupled with facilitated referral of infants to a health centre (n=2007)	Control birth attendants continued their existing standard of care (basic obstetric skills and use of clean delivery kits). (n=1552)	2.5 years	Stillbirths, neonatal deaths, perinatal deaths, and other care related outcomes	Selection=LR<br>Performance and detection = LR<br>Attrition=LR<br>Reporting = LR
Okeibunor 2011 [19]	Nigeria	Women (n=1372)	**Enhanced Perinatal care/education**<br>Under the programme, volunteer community-directed distributors (CDDs) trained to deliver ITNs and IPTp drugs as well as basic counselling services to pregnant women (n=751)	Standard care (n=621)	Not Reported	Care related outcomes	Selection=UR<br>Performance and detection = UR<br>Attrition=UR<br>Reporting = LR
Tripathy 2010 [20]<br><br>**ISRCTN21817853**	India	18207 live births	**Community mobilization**<br>Implemented a participatory learning cycle, through developing women's groups where they identify and prioritize maternal and newborn health problems in their community, collectively selected	Health committees in control clusters were formed to give Community a voice in the design and management of local	2 years	Maternal and neonatal health care seeking, neonatal deaths, stillbirths, perinatal deaths,	Selection=UR<br>Performance and detection = HR<br>Attrition=LR<br>Reporting = LR

Author/year	Country	Target population	Intervention	Comparison	Duration of intervention	Outcomes reported	Assessment of bias
			relevant strategies to address those problems, implemented the strategies, and evaluated the results (n=9770)	health services (n=9620)		maternal deaths, and other care related outcomes	
Azad 2010 [21]<br>**ISRCTN54792066**	Bangladesh	29889 live births	**Community mobilization**<br>Implemented a participatory learning and action cycle in which they identify and prioritize problems, then formulate strategies and lastly implemented and monitored and finally evaluated the process +intervention group was again divided into two according to the trained TBAs for asphyxia or not (n=20943)	Control group was not provided with participatory learning groups (n=22774)	2 years	Neonatal health care seeking, neonatal deaths, stillbirths, perinatal deaths, maternal deaths, and other care related outcomes	Selection=LR<br>Performance and detection = HR<br>Attrition=LR<br>Reporting = LR
Darmstadt 2010 [22, 23]<br>**NCT00198627**	Bangladesh	A total of 4616 and 5241 live births were recorded from 9987 and 11153 participants	**Home visitation**<br>CHWs identified pregnant women; made two antenatal home visits to promote birth and newborn care preparedness; made four postnatal home visits to negotiate preventive care practices and to assess newborns for illness; and referred sick neonates to a hospital and facilitated compliance. (n=4616 live births)	Standard care (n=5241 live births)	3 years	Neonatal deaths, stillbirths, perinatal deaths, other care related and health care seeking outcomes	Selection=LR<br>Performance and detection = LR<br>Attrition=LR<br>Reporting = LR
Midhet 2010 [24]	Pakistan	1661 pregnant women	**Community mobilization**<br>The IEEC for women was designed to increase awareness of safe motherhood and neonatal health<br>To women only (n=836)<br>**Community mobilization**<br>To couples (n=703)	Standard care (n=1022)	4 years	Maternal health care seeking, neonatal mortality, stillbirths, Perinatal deaths, Maternal deaths, and other care related outcomes	Selection=LR<br>Performance and detection = UR<br>Attrition=UR<br>Reporting = LR
Kumar 2008 [25-28]<br>**NCT00198653**	India	Population 104,123	**Community mobilization and home visitation**<br>Home visitation and community empowerment to promote essential newborn (n=1625)<br>**Community mobilization and home visitation**<br>Home visitation and community empowerment to promote essential newborn + thermospot (n=1175)	A control group received the usual services (n=1173)	16 months	Maternal and neonatal health care seeking, maternal deaths, neonatal deaths, stillbirths, perinatal deaths, and other care related outcomes	Selection=LR<br>Performance and detection = LR<br>Attrition=LR<br>Reporting = LR
Bhutta 2008 [29, 30]<br>**ISRCTN16247511**	Pakistan	Women of reproductive age	**Community mobilization and home visitation**<br>Home-based newborn care and community organization and mobilization and group education sessions to establish an emergency transport fund for mothers and newborns (n=1478)	LHW training in home based newborn care (n=1401)	24 months	Neonatal deaths, stillbirths, perinatal deaths and other care related outcomes	Selection=UR<br>Performance and detection = LR<br>Attrition=UR<br>Reporting = LR

Author/year	Country	Target population	Intervention	Comparison	Duration of intervention	Outcomes reported	Assessment of bias
Sloan 2008 [31]	Bangladesh	Women of reproductive age (4165 live births)	**Enhanced Perinatal care/education**<br>Trained community workers to teach CKMC to expectant mothers (n=2080)	No CKMC care education (n=2003)	6 months	Neonatal health care seeking, neonatal deaths, infant deaths, and other care related outcomes	Selection=LR<br>Performance and detection = HR<br>Attrition=LR<br>Reporting = LR
Bashour 2008 [32]	Syria	876 women	**Home visitation**<br>Registered midwives with special training made a one or a series of home visits providing information, educating, and supporting women 4 postnatal home visits (n=285)	no visit (n=301)	4 months	Care related outcomes, maternal health care seeking	Selection=HR<br>Performance and detection = HR<br>Attrition=UR<br>Reporting = LR
			**Home visitation**<br>One home visit (n=294)				
Mboyne 2008 [33]	Uganda	Pregnant women	**Enhanced Perinatal care/education**<br>Fifty-one CHWs were trained for dangers of malaria in pregnancy; malaria-prevention interventions; the benefits and side-effects of SP; taking blood samples for parasite count; haemoglobin analysis; taking the baby's weight; and estimating the gestational age (n=2081)	Routine delivery of IPTp (n=704)	Not Reported	Care related outcomes	Selection=LR<br>Performance and detection = UR<br>Attrition=UR<br>Reporting = LR
Baqui 2008 [34]<br><br>**NCT00198705**	Bangladesh	19557 pregnant women<br>19525 deliveries	**Home visitation**<br>Trained all TBAs for improved services for enhanced referrals, ANC and postpartum visits, and provided them with delivery kits. TBAs were also linked with LHWs in the community (n=14880)	TBAs were not trained and did not receive delivery kits. Routine care was delivered by LHWs (n=15779)	2 years	Neonatal deaths, stillbirths, perinatal deaths and other care related outcomes	Selection=LR<br>Performance and detection = HR<br>Attrition=LR<br>Reporting = LR
			**Community mobilization**<br>Community mobilization (n=16499)				
Mullany 2007 [35]	Nepal	442 pregnant women	**Enhanced Perinatal care/education**<br>Education with husband antenatal health education (n=133)	No education at all (n=149)	One session of education	Care related outcomes	Selection=LR<br>Performance and detection = UR<br>Attrition=UR<br>Reporting = LR
			**Enhanced Perinatal care/education**<br>Couple education – antenatal health education (n=130)				
Bari 2006 [36]	Bangladesh	3228 deliveries	**Home visitation**<br>Promoting care-seeking for sick newborns through health education of families, identification and referral of sick newborns in the community by CHWs, and strengthening of neonatal care (n=520)	Standard care (n=548)	2 years	Neonatal health care seeking, and other care related outcomes	Selection=UR<br>Performance and detection = UR<br>Attrition=UR<br>Reporting = LR
Jokhio 2005 [37]	Pakistan	19559 women	**Enhanced Perinatal care/education**	Usual care (n=9443)	6 months	Maternal deaths, neonatal deaths,	Selection=UR

Author/year	Country	Target population	Intervention	Comparison	Duration of intervention	Outcomes reported	Assessment of bias
			Trained TBAs to provide antepartum, intrapartum, and postpartum care; clean delivery; refer women EmONC (n=10114)			stillbirths, perinatal deaths, and other care related outcomes	Performance and detection = LR<br>Attrition=UR<br>Reporting = LR
Manandhar 2004 [38-41]<br><br>**ISRCTN31137309**	Nepal	Women of reproductive age	**Community mobilization**<br>Participatory learning skills and generated information on pregnancy and childbirth, covering beliefs and practices in both uncomplicated and complicated pregnancies (n=3036).	improvements in equipment and training provided at all levels of the healthcare System (n=3344).	2 years	Maternal and neonatal health care seeking, maternal deaths, neonatal deaths, stillbirths, perinatal deaths, maternal deaths, and other care related outcomes	Selection=LR<br>Performance and detection = HR<br>Attrition=LR<br>Reporting = LR
Srinivasan 1995 [42]	India	1623 pregnancies	**Enhanced Perinatal care/education**<br>In high risk intervention package group trained midwives identified high-risk pregnancies and intervened accordingly TNG intervention package group does not include identification of high risk pregnancies (n=573)	Received general health services and no special inputs were provided by project staff (n=594)	3 years	Care related outcomes	Selection=UR<br>Performance and detection = UR<br>Attrition=UR<br>Reporting = LR
Abbreviations= ANC: Antenatal Care; CKMC: Community Kangaroo Mother Care; CHW: Community Health Worker; EmONC: Emergency Obstetric and Neonatal Care; HR: High Risk; IEEC: Information, Education, Empowerment and Communication; ITN: Insecticide Treated Bednets; IPTp: Intermittent Preventive Treatment in Pregnancy; LHW: Lady Health Worker; LR: Low Risk; MTCT: Mother To Child Transmission; n=number; PMTCT: Prevention of Mother to Child Transmission; TBA: Traditional Birth Attendants; UC: Unclear							

Characteristics of included studies: non-RCTs

Author/year	Country	Target population	Intervention	Comparison	Duration of intervention	Outcomes reported	Assessment of risk of bias
Foord 1995[43]	Gambia	1449 pregnancies	ANC, provided malaria prophylaxis, identification of infections and early referral to facilities (n=780)	Standard care (n=669)	3 years	Stillbirths, perinatal deaths, and care related outcomes	Selection=HR<br>Performance and detection = UR<br>Attrition=UR<br>Reporting = LR
Mann 2010[44]	India	Women were eligible if they were between 15 and 59 years of age	Community-centred primary health care. Activities in the project villages are carried out at three levels: village health workers, mobile health teams and the secondary-care hospital in Jamkhed (n=3002)	Standard care (n=3003)	15 years	Neonatal deaths, infant deaths, under 5 deaths, and other care related outcomes	Selection=UR<br>Performance and detection = UR<br>Attrition=UR<br>Reporting = LR
Turan 2011[45]	Eritrea	postpartum women	participatory educational sessions on safe motherhood topics with women and men in the community	Standard care	2 years	Care related outcomes	Selection=UR<br>Performance and detection = UR<br>Attrition=UR<br>Reporting = LR

Xu 1995[46]	China	Pregnant women	Improved access to maternal care services, mass health education and strengthened obstetric emergency services	Standard care	3 years	Maternal deaths, and other care related outcomes	Selection=UR<br>Performance and detection = UR<br>Attrition=UR<br>Reporting = LR
Zhang 2004[47]	China	Pregnant women (n=348)	Reproductive health and family planning project	-	4 years	Care related outcomes	Selection=UR<br>Performance and detection = UR<br>Attrition=UR<br>Reporting = LR
Alisjahbana 1995 [48]	Indonesia	3275 pregnancies	Trained TBAs for enhanced complication referrals, teaching mothers for danger signs. Improved accessibility to health care services and trained hospital doctors and nurses for appropriate care management, distributed home based maternal and neonatal action records (n=2275)	Routine services provided by government health care facilities and hospitals (n=1000)	1 year	Perinatal deaths, and other care related outcomes	Selection=HR<br>Performance and detection = HR<br>Attrition=UR<br>Reporting = LR
Bang 1999 [49-52]	India	5921 live births	Train paramedics, village HCW, and TBA in administration of antibiotics and counseling in mother and newborn care (n=979)	In control areas these tasks were done by the government health services and the Integrated Child Development Service (ICDS) workers (n=1108)	3 years	Stillbirths, perinatal deaths, neonatal deaths, and other care related outcomes	Selection=HR<br>Performance and detection = UR<br>Attrition=UR<br>Reporting = LR
Baqui 2008 Care India [53, 54]	India	13826 live births	Antenatal intervention, birth preparedness, disposable delivery kit, newborn care, postnatal intervention vs. routine care (n=7918)	Received standard government health and Integrated Child Development Services (n=6014)	2.5 years	Neonatal deaths, and other care related outcomes	Selection=HR<br>Performance and detection = LR<br>Attrition=UR<br>Reporting = UR
Ronsmans 1997[55]	Bangladesh	24059 live births	MCH-FP areas (referrals for sick cases, safe delivery kit, iron and folate for mothers, family planning, management of obstetric complication etc.) (n=2615)	Comparison area did not have MCH-FP services and was provided with routine services (n=2992)	6 years	Maternal deaths	Selection=HR<br>Performance and detection = UR<br>Attrition=UR<br>Reporting = UR
Bang 2005 [56]	India	5651 deliveries<br><br>5510 live births	Assessed the impact of TBA training on neonatal resuscitation and home based care education on neonatal mortality (n=2512)	TBAs in control areas were not additionally trained as TBAs in intervention arm, but they did receive usual training from government sources (n=2958)	10 years	Care related outcomes	Selection=HR<br>Performance and detection = UR<br>Attrition=UR<br>Reporting = LR
Greenwood 1990[57]	Gambia	1963 pregnancies<br><br>1843 live births	Government of Gambia implemented OHC service and trained TBAs regarding clean deliveries at home, referrals for delivery and promotion of antenatal and post care among mothers (n=1208)	Non- PHC areas have routine delivery service outlets like health facilities and hospitals (n=705)	5 years	Perinatal deaths, neonatal deaths, and other care related outcomes	Selection=HR<br>Performance and detection = UR<br>Attrition=UR<br>Reporting = UR
Syed 2006 [58]	Bangladesh	3110 women	Essential newborn care was integrated into the ongoing	Standard care (n=323)	2 years	Care related outcome	Selection=UR<br>Performance and detection = UR

			interventions of the NGOs. The major components of the interventions included: increasing the coverage of health workers and community-based caregivers trained and competent in providing essential newborn care and promoting positive maternal and newborn-care practices (n=2787)				Attrition=UR<br>Reporting = LR
Hounton 2008 [23, 59-62]	Burkina Faso	40446 women	Effects of the Skilled Care Initiative (SCI) community mobilisation on pregnancy (N=18658)	Standard care (n=21788)	2 years	Maternal deaths	Selection=HR<br>Performance and detection = UR<br>Attrition=UR<br>Reporting = LR
Liu 2010 [63]	China	Pregnant women (n=9620)	Establishing the emergency obstetric centres, Strengthening the maternal health-service quality in hospitals, changing the functions of village doctors, so that they can replace the midwife (n=2991)	Standard care (n=6629)	4 years	Care related outcomes	Selection=HR<br>Performance and detection = UR<br>Attrition=UR<br>Reporting = LR
Fauveau 1991 [64]	Bangladesh	Women of reproductive age (n=21824)	midwives were equipped to treat immediately obstetric complications at their onset, and were backed up by an effective chain of referral. (n=1534 live births)	Standard care (n=1784)	3 years	Maternal deaths	Selection=HR<br>Performance and detection = UR<br>Attrition=UR<br>Reporting = LR
Abbreviations= ANC: Antenatal Care; HCW: Health Care Worker; HR: High Risk; LR: Low Risk; MCH-FP: Maternal, Child Health and Family Planning; MNH: Maternal and Newborn Health; n=number; NGO: Non-Government Organization; OHC: Outreach Health Centre; TBA: Traditional Birth Attendants; UR: Unclear Risk							

Characteristics of included studies: (before/after, interrupted time series) studies

Study ID	Country	Study design	Participants (n)	Intervention	Outcomes reported	Assessment of bias
Awasthi 2009 [65-70]	India	Before/After study	Newborns born in last 48 hours (n=490)	Intervention comprised of Neonatal Well-Being Card. The card contained pictorial representation of neonatal danger signs, and messages regarding the importance of qualified medical care-seeking	Qualified medical care-seeking for all Illnesses	Selection=HR<br>Performance and detection = LR<br>Attrition=LR<br>Reporting = LR<br>Baseline outcome and characteristics = LR
Daga 1993 [71]	India	Before/after study	Children under 6 years of age	Training of dais in newborn care which included warmth, resuscitation and identification and referral of a baby. Training of anganwadi workers to make a home visit, referring a baby with foot length less than 6.5 cm to PHC if dai fails to make correct assessment, monitor the progress of a borderline preterm/low birth weight baby	ANC registration, newborns referrals, neonatal deaths	Selection=UR<br>Performance and detection = UR<br>Attrition=UR<br>Reporting = LR<br>Baseline outcome and characteristics = UR
Emond 2002 [72]	Brazil	Before/after study	Births Pre-intervention 1195<br>Births- post interventions 1225	Women attending the community antenatal clinics were assessed as high or low risk. High-risk pregnancies were booked for delivery at hospital, Low-risk mothers were offered delivery at the polyclinic. In	Stillbirths/miscarriages, early neonatal deaths, prematurity, low birth	Selection=UR<br>Performance and detection = UR<br>Attrition=UR<br>Reporting = LR

Study ID	Country	Study design	Participants (n)	Intervention	Outcomes reported	Assessment of bias
				addition, social workers from the health centres, who also made a limited number of home visits.	weight, caesarean births, delivery at community clinic	Baseline outcome and characteristics = UR
Fujino 2009[73]	Zambia (low income areas)	ITS	1546 mothers	There was a perception among the Dominican nurses that women delayed accessing care because they held beliefs and attitudes regarding obstetric care that caused them to delay seeking help.	Response to danger signs	Selection=UR<br>Performance and detection = UR<br>Attrition=LR<br>Reporting = LR<br>Baseline outcome and characteristics = UR
Garces 2012[74]	Guatemala (rural clusters)	Before/after studies	522 TBAs, 10 Physicians and 12 nurse/nurse midwives participated in the study	Training of practicing birth attendants within each community to evaluate clinical condition of newborns, and Apgar scores. Birth attendants included TBAs, nurses, nurse-midwives, and physicians.	Perinatal deaths, stillbirth, early neonatal mortality	Selection=UR<br>Performance and detection = UR<br>Attrition=UR<br>Reporting = LR<br>Baseline outcome and characteristics = UR
Saaka 2011[75]	Ghana	Before/after intervention	young children (6–59 months) and women of childbearing age (15–45 years). Only women aged 15–45 years, whose children were aged 0–36 months	Promoting infant feeding practices, health-seeking behaviours at the community level and enhancing the quality of care, effective case management of common childhood illnesses, EPI, improving geographical access to health services by supporting the construction of improved food storage facilities and growth monitoring centres, support of community-level health promotion activities including support of mothers' support groups and holding of health campaigns, growth monitoring, etc.	Underweight, acute and chronic malnutrition, initiation of breastfeeding, exclusive breastfeeding, continued breastfeeding, fully immunized children, health-seeking behaviour for ARI	Selection=UR<br>Performance and detection = UR<br>Attrition=UR<br>Reporting = LR<br>Baseline outcome and characteristics = UR
Thassari 2000[76]	Thailand	Before/after study	pregnant women who attended an ANC clinic	Preparation for pregnancy, nutrition during pregnancy and breast feeding periods, breast feeding, preparation for delivery and preparation for postpartum.	Change in behavior, satisfaction	Selection=UR<br>Performance and detection = UR<br>Attrition=UR<br>Reporting = LR<br>Baseline outcome and characteristics = UR
O'rourke 1998[77]	Bolivia	Before/after study	Not mentioned	Impact of women group diagnosing, designing, implementing, and evaluating community-based solution to maternal and perinatal health problems	Perinatal mortality, breastfeeding rates	Selection=UR<br>Performance and detection = UR<br>Attrition=UR<br>Reporting = LR<br>Baseline outcome and characteristics = UR
Moran 2006 [78]	Burkina Faso	Before/after study	180 pregnant women and 180 women delivered in 12 months	MNH program of JPIEGO focused on birth preparedness, recognition of danger sings	Planning for delivery with skilled birth attendance	Selection=UR<br>Performance and detection = UR<br>Attrition=UR<br>Reporting = LR<br>Baseline outcome and characteristics = UR
Dongre 2009 [79-84]	India	Before/after study	Not mentioned	Educate women about newborn danger sings birth preparedness, health care seeking, and conduction of monthly village based meeting	Planning for delivery with skilled birth attendance	Selection=UR<br>Performance and detection = UR<br>Attrition=UR<br>Reporting = LR

Study ID	Country	Study design	Participants (n)	Intervention	Outcomes reported	Assessment of bias
						Baseline outcome and characteristics = UR
Carlo 2010 [85-87]	Argentina, Congo DR, India, Zambia Guatemala, and Pakistan	Before/after study	57,643 infants	Essential Newborn Care included routine neonatal care, initiation of breathing and resuscitation (including bag and mask ventilation), thermoregulation, early and exclusive breast-feeding, kangaroo (skin-to-skin) care, care of small babies, recognition of danger signs, and recognition and initial management of complications.	Neonatal deaths, stillbirths and perinatal deaths	Selection=LR<br>Performance and detection = LR<br>Attrition=LR<br>Reporting = LR<br>Baseline outcome and characteristics = UR
Coskun 2009[88]	Turkey	Evaluation study		Antenatal period education module: Adaptation to pregnancy and health in pregnancy, Preparation for childbirth, Labour and delivery pain management, Maternity ward/Immediately after delivery, Puerperal women's health	Iron/folate, breastfeeding	Selection=UR<br>Performance and detection = UR<br>Attrition=UR<br>Reporting = LR<br>Baseline outcome and characteristics = UR
Hodgins 2010 [89]	Nepal	Before/after study	1470 each before & after intervention recently delivered women	community-based antenatal counseling and dispensing and an early postnatal home visit; most activities were carried out by community-based health volunteers	Advice on arrange skilled birth attendant, newborn danger signs, delivery related danger signs, delayed bathing, delay in care seeking	Selection=LR<br>Performance and detection = LR<br>Attrition=UR<br>Reporting = LR<br>Baseline outcome and characteristics = UR
Mushi 2010 [90]	Tanzania	Before/after study	512 deliveries	The intervention package comprised of two main components (a) training of Safe Motherhood Promoters (these were trained specifically for this intervention study) and (b) education and awareness on maternal health aspects.	Deliveries with skilled attendant, Early ANC booking	Selection=UR<br>Performance and detection = UR<br>Attrition=UR<br>Reporting = LR<br>Baseline outcome and characteristics = UR
Abbreviations= ANC: Antenatal Care; ARI: Acute Respiratory Infections; EPI: Expanded Program on Immunization; LR: Low Risk; MNH: Maternal and Newborn Health; n:number; TBA: Traditional Birth Attendants; UR: Unclear Risk						

Web Annex 2:

Neonatal Mortality

Study or Subgroup	log[Risk Ratio]	SE	Intervention Total	Control Total	Weight	Risk Ratio IV, Random, 95% CI
Azad 2010	-0.1054	0.1068	4457	4441	4.4%	0.90 [0.73, 1.11]
Baqui 2008 (1)	-0.4155	0.1732	2812	1436	3.4%	0.66 [0.47, 0.93]
Baqui 2008 (2)	-0.0513	0.1632	3009	1436	3.6%	0.95 [0.69, 1.31]
Bashour 2008 (3)	4.0877	1.4238	293	148	0.1%	59.60 [3.66, 970.97]
Bashour 2008 (4)	1.9872	1.5096	284	148	0.1%	7.30 [0.38, 140.61]
Bhandari 2012	-0.0943	0.0657	29667	30813	4.9%	0.91 [0.80, 1.04]
Bhutta 2008	-0.3704	0.1181	2932	2610	4.2%	0.69 [0.55, 0.87]
Bhutta 2011	-0.1324	0.0601	12028	11005	4.9%	0.88 [0.78, 0.99]
Colbourn 2013	-0.1054	0.093	10055	9931	4.5%	0.90 [0.75, 1.08]
Darmstadt 2010	-0.1393	0.1257	4616	5214	4.1%	0.87 [0.68, 1.11]
Fottrell 2013	-0.4308	0.1339	8819	9896	4.0%	0.65 [0.50, 0.85]
Gill 2011	-0.5978	0.2606	1889	1466	2.4%	0.55 [0.33, 0.92]
Jokhio 2005	-0.3425	0.0692	340	439	4.8%	0.71 [0.62, 0.81]
Kirkwood 2013	-0.0943	0.1562	7721	7898	3.7%	0.91 [0.67, 1.24]
Kumar 2008 (5)	-0.7765	0.1394	1522	539	3.9%	0.46 [0.35, 0.60]
Kumar 2008 (6)	-0.734	0.1612	1087	540	3.6%	0.48 [0.35, 0.66]
Lewycka 2013 (7)	-0.3147	0.1931	4610	1653	3.1%	0.73 [0.50, 1.07]
Lewycka 2013 (8)	-0.2744	0.1839	4680	1654	3.3%	0.76 [0.53, 1.09]
Lewycka 2013 (9)	-0.0726	0.1828	4494	1653	3.3%	0.93 [0.65, 1.33]
Manandhar 2004	-0.3567	0.1419	2899	3226	3.9%	0.70 [0.53, 0.92]
Midhet 2010 (10)	-0.393	0.0634	740	448	4.9%	0.68 [0.60, 0.76]
Midhet 2010 (11)	-0.4529	0.0697	622	447	4.8%	0.64 [0.55, 0.73]
More 2012	0.3507	0.184	7944	7759	3.3%	1.42 [0.99, 2.04]
Persson 2013	-0.0408	0.1397	11818	10559	3.9%	0.96 [0.73, 1.26]
Sloan 2008	0.0583	0.1691	2121	2044	3.5%	1.06 [0.76, 1.48]
Tripathy 2010	-0.3711	0.0713	9388	8819	4.8%	0.69 [0.60, 0.79]
Wu 2011	0.365	0.081	2094	2062	4.7%	1.44 [1.23, 1.69]
**Total (95% CI)**			**142941**	**128284**	**100.0%**	**0.80 [0.72, 0.89]**

Heterogeneity: Tau² = 0.06; Chi² = 149.64, df = 26 (P < 0.00001); I² = 83%
Test for overall effect: Z = 4.11 (P < 0.0001)

Risk Ratio
IV, Random, 95% CI

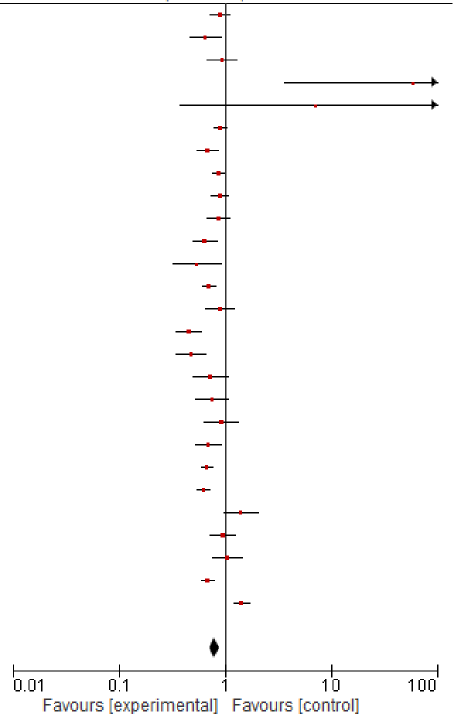

Footnotes
(1) Home Care
(2) Community Care
(3) Single visit
(4) Four visits
(5) Essential Newborn Care
(6) Essential Newborn Care + thermo spot
(7) Home visitation
(8) Community mobilization
(9) Community mobilization + home visitation
(10) Women - Information and Education for Empowerment and Change
(11) Couple - Information and Education for Empowerment and Change

Web annex 3
Search Strategy

[(‘care seeking’ OR ‘care-seeking’ OR ‘health care’ OR ‘delivery of health care’ [MEsH Terms] OR ‘health care seeking’ OR ‘community based intervention*’ OR ‘community-based intervention*’) AND (mother* OR mother [MEsH Terms] OR maternal OR women OR newborn* OR neonat*OR "infant, newborn"[MeSH Terms])]

References

1. Acharya A, Lalwani T, Dutta R, Rajaratnam JK, Ruducha J, Varkey LC, Wunnava S, Menezes L, Taylor C, Bernson J: **Evaluating a Large-Scale Community-Based Intervention to Improve Pregnancy and Newborn Health Among the Rural Poor in India**. *Am J Public Health* 2015, **105**:144-152.
2. Fottrell E, Azad K, Kuddus A, Younes L, Shaha S, Nahar T, Aumon BH, Hossen M, Beard J, Hossain T *et al*: **The Effect of Increased Coverage of Participatory Women's Groups on Neonatal Mortality in Bangladesh: A Cluster Randomized Trial**. *JAMA Pediatr* 2013, **167**(9):816-825.
3. Persson L, Nga NT, Malqvist M, Hoa DTP, Eriksson L, Wallin L, Selling K, Huy TQ, Duc DM, Tiep TV: **Effect of facilitation of local maternal-and-newborn stakeholder groups on neonatal mortality: cluster-randomized controlled trial**. *PLoS Med* 2013, **10**(5):e1001445.
4. Lewycka S, Mwansambo C, Rosato M, Kazembe P, Phiri T, Mganga A, Chapota H, Malamba F, Kainja E, Newell ML *et al*: **Effect of women's groups and volunteer peer counselling on rates of mortality, morbidity, and health behaviours in mothers and children in rural Malawi (MaiMwana): a factorial, cluster-randomised controlled trial**. *Lancet* 2013, **381**(9879):1721-1735.
5. Lewycka S, Mwansambo C, Kazembe P, Phiri T, Mganga A, Rosato M, Chapota H, Malamba F, Vergnano S, Newell ML *et al*: **A cluster randomised controlled trial of the community effectiveness of two interventions in rural Malawi to improve health care and to reduce maternal, newborn and infant mortality.** *Trials* 2010, **11**:88.
6. Kirkwood BR, Manu A, ten Asbroek AH, Soremekun S, Weobong B, Gyan T, Danso S, Amenga-Etego S, Tawiah-Agyemang C, Owusu-Agyei S *et al*: **Effect of the Newhints home-visits intervention on neonatal mortality rate and care practices in Ghana: a cluster randomised controlled trial**. *Lancet* 2013, **381**(9884):2184-2192.
7. Nsibande d, Doherty T, Ijumba P, Tomlinson M, Jackson D, Sanders D, Lawn J: **Assessment of the uptake of neonatal and young infant referrals by community health workers to public health facilities in an urban informal settlement, KwaZulu-Natal, South Africa. Nsibande et al.** *BMC Health Services Research* 2013, **14**:47.
8. Tomlinson M, Doherty T, Jackson D, Lawn JE, Ijumba P, Colvin M, Nkonki L, Daviaud E, Goga A, Sanders D: **An effectiveness study of an integrated, community-based package for maternal, newborn, child and HIV care in South Africa: study protocol for a randomized controlled trial**. *Trials* 2011, **12**(1):236.

9. Tomlinson M, Doherty T, Ijumba P, Jackson D, Lawn J, Persson LÅ, Lombard C, Sanders D, Daviaud E, Nkonki L *et al*: **Goodstart: a cluster randomised effectiveness trial of an integrated, community-based package for maternal and newborn care, with prevention of mother-to-child transmission of HIV in a South African township.** *Trop Med Int Health* 2014, **19**(3):256-266.
10. Magoma M, Requejo J, Campbell O, Cousens S, Merialdi M, Filippi V: **The effectiveness of birth plans in increasing use of skilled care at delivery and postnatal care in rural Tanzania: a cluster randomised trial**. *Trop Med Int Health* 2013, **18**(4):435-443.
11. More NS, Bapat U, Das S, Alcock G, Patil S, Porel M, Vaidya L, Fernandez A, Joshi W, Osrin D: **Community mobilization in Mumbai slums to improve perinatal care and outcomes: a cluster randomized controlled trial**. *PLoS Med* 2012, **9**(7):e1001257.
12. More NS, Bapat U, Das S, Patil S, Porel M, Vaidya L, Koriya B, Barnett S, Costello A, Fernandez A: **Cluster-randomised controlled trial of community mobilisation in Mumbai slums to improve care during pregnancy, delivery, postpartum and for the newborn**. *Trials* 2008, **9**(7).
13. Colbourn T, Nambiar B, Bondo A, Makwenda C, Tsetekani E, Makonda-Ridley A, Msukwa M, Barker P, Kotagal U, Williams C: **Effects of quality improvement in health facilities and community mobilization through women's groups on maternal, neonatal and perinatal mortality in three districts of Malawi: MaiKhanda, a cluster randomized controlled effectiveness trial**. *Int Health* 2013, **5**(3):180-195.
14. Bhandari N, Mazumder S, Taneja S, Sommerfelt H, Strand TA: **Effect of implementation of Integrated Management of Neonatal and Childhood Illness (IMNCI) programme on neonatal and infant mortality: cluster randomised controlled trial**. *BMJ* 2012, **344**.
15. Bhutta ZA, Soofi S, Cousens S, Mohammad S, Memon ZA, Ali I, Feroze A, Raza F, Khan A, Wall S *et al*: **Improvement of perinatal and newborn care in rural Pakistan through community-based strategies: a cluster-randomised effectiveness trial**. *Lancet* 2011, **377**(9763):403-412.
16. Soofi S: **Impact of an intervention package on perinatal and neonatal mortality delivered through lady health workers (LHWs) and traditional birth attendants (Dais) in rural Pakistan**. In: *Pediatric Academic Societies Annual Meeting: 2010 May 1-4; Vancouver, Canada*; 2010 May 1-4.
17. Wu Z, Viisainen K, Wang Y, Hemminki E: **Evaluation of a community-based randomized controlled prenatal care trial in rural China**. *BMC Health Services Research* 2011, **11**(1):92.
18. Gill CJ, Phiri-Mazala G, Guerina NG, Kasimba J, Mulenga C, MacLeod WB, Waitolo N, Knapp AB, Mirochnick M, Mazimba A: **Effect of training traditional birth attendants on neonatal mortality (Lufwanyama Neonatal Survival Project): randomised controlled study**. *BMJ* 2011, **342**.
19. Okeibunor JC, Orji BC, Brieger W, Ishola G, Otolorin E, Rawlins B, Ndekhedehe EU, Onyeneho N, Fink G: **Preventing malaria in pregnancy through community-directed interventions: evidence from Akwa Ibom State, Nigeria**. *Malar J* 2011, **10**:227.
20. Tripathy P, Nair N, Barnett S, Mahapatra R, Borghi J, Rath S, Rath S, Gope R, Mahto D, Sinha R: **Effect of a participatory intervention with women's groups on birth outcomes and maternal depression in Jharkhand and Orissa, India: a cluster-randomised controlled trial**. *Lancet* 2010, **375**(9721):1182-1192.
21. Azad K, Barnett S, Banerjee B, Shaha S, Khan K, Rego AR, Barua S, Flatman D, Pagel C, Prost A: **Effect of scaling up women's groups on birth outcomes in three rural districts in Bangladesh: a cluster-randomised controlled trial**. *Lancet* 2010, **375**(9721):1193-1202.
22. Darmstadt GL, Choi Y, Arifeen SE, Bari S, Rahman SM, Mannan I, Seraji HR, Winch PJ, Saha SK, Ahmed AS *et al*: **Evaluation of a cluster-randomized controlled trial of a package of community-based maternal and newborn interventions in Mirzapur, Bangladesh**. *PLoS One* 2010, **5**(3):e9696.

23. Darmstadt GL, El Arifeen S, Choi Y, Bari S, Rahman SM, Mannan I, Winch P, Ahmed ASMNU, Seraji HR, Begum N: **Household surveillance of severe neonatal illness by community health workers in Mirzapur, Bangladesh: coverage and compliance with referral**. *Health policy and planning* 2010, **25**(2):112-124.
24. Midhet F, Becker S: **Impact of community-based interventions on maternal and neonatal health indicators: Results from a community randomized trial in rural Balochistan, Pakistan**. *Reprod Health* 2010, **7**:30.
25. Kumar V, Mohanty S, Kumar A, Misra RP, Santosham M, Awasthi S, Baqui AH, Singh P, Singh V, Ahuja RC *et al*: **Effect of community-based behaviour change management on neonatal mortality in Shivgarh, Uttar Pradesh, India: a cluster-randomised controlled trial**. *Lancet* 2008, **372**(9644):1151-1162.
26. Kumar V, Kumar A, Das V, Srivastava NM, Baqui AH, Santosham M, Darmstadt GL: **Community-driven impact of a newborn-focused behavioral intervention on maternal health in Shivgarh, India**. *int J Gynaecol Obstet* 2012, **117**(1):48-55.
27. Willis JR, Kumar V, Mohanty S, Singh P, Singh V, Baqui AH, Awasthi S, Singh JV, Santosham M, Darmstadt GL: **Gender differences in perception and care-seeking for illness of newborns in rural Uttar Pradesh, India**. *J Health Popul Nutr* 2009, **27**(1):62-71.
28. Darmstadt GL, Kumar V, Yadav R, Singh V, Singh P, Mohanty S, Baqui AH, Bharti N, Gupta S, Misra RP: **Introduction of community-based skin-to-skin care in rural Uttar Pradesh, India**. *Journal of Perinatology* 2006, **26**(10):597-604.
29. Bhutta ZA, Ahmed T, Black RE, Cousens S, Dewey K, Giugliani E, Haider BA, Kirkwood B, Morris SS, Sachdev HPS: **What works? Interventions for maternal and child undernutrition and survival**. *Lancet* 2008, **9610**(3):417-440.
30. Bhutta Z, Soofi S, Memon Z, Mohammad S, Khan A, Cousens., al e: **Community mobilisation with women's groups facilitated by Accredited Social Health Activists (ASHAs) to improve maternal and newborn health in underserved areas of Jharkhand and Orissa: Study protocol for a cluster-randomised controlled trial**. In: *Pediatric Academic Societies Annual Meeting: 2009 May 2-5; Baltimore, Maryland, USA*; 2009 May 2-5.
31. Sloan NL, Ahmed S, Mitra SN, Choudhury N, Chowdhury M, Rob U, Winikoff B: **Community-based kangaroo mother care to prevent neonatal and infant mortality: a randomized, controlled cluster trial**. *Pediatrics* 2008, **121**(5):e1047-1059.
32. Bashour HN, Kharouf MH, Abdulsalam AA, El Asmar K, Tabbaa MA, Cheikha SA: **Effect of postnatal home visits on maternal/infant outcomes in Syria: a randomized controlled trial**. *Public Health Nurs* 2008, **25**(2):115-125.
33. Mbonye AK, Schultz Hansen K, Bygbjerg IC, Magnussen P: **Effect of a community-based delivery of intermittent preventive treatment of malaria in pregnancy on treatment seeking for malaria at health units in Uganda**. *Public Health* 2008, **122**(5):516-525.
34. Baqui AH, El-Arifeen S, Darmstadt GL, Ahmed S, Williams EK, Seraji HR, Mannan I, Rahman SM, Shah R, Saha SK: **Effect of community-based newborn-care intervention package implemented through two service-delivery strategies in Sylhet district, Bangladesh: a cluster-randomised controlled trial**. *Lancet* 2008, **371**(9628):1936-1944.
35. Mullany BC, Becker S, Hindin MJ: **The impact of including husbands in antenatal health education services on maternal health practices in urban Nepal: results from a randomized controlled trial**. *Health Educ Res* 2007, **22**(2):166-176.
36. Bari S, Mannan I, Rahman MA, Darmstadt GL, Serajil MH, Baqui AH, El Arifeen S, Rahman SM, Saha SK, Ahmed AS *et al*: **Trends in use of referral hospital services for care of sick newborns in a community-based intervention in Tangail District, Bangladesh**. *J Health Popul Nutr* 2006, **24**(4):519-529.

37. Jokhio AH, Winter HR, Cheng KK: **An intervention involving traditional birth attendants and perinatal and maternal mortality in Pakistan**. *N Engl J Med* 2005, **352**(20):2091-2099.
38. Manandhar DS, Osrin D, Shrestha BP, Mesko N, Morrison J, Tumbahangphe KM, Tamang S, Thapa S, Shrestha D, Thapa B *et al*: **Effect of a participatory intervention with women's groups on birth outcomes in Nepal: cluster-randomised controlled trial**. *Lancet* 2004, **364**(9438):970-979.
39. Morrison J, Tamang S, Mesko N, Osrin D, Shrestha B, Manandhar M, Manandhar D, Standing H, Costello A: **Women's health groups to improve perinatal care in rural Nepal**. *BMC Pregnancy and Childbirth* 2005, **5**(1):6.
40. Osrin D, Mesko N, Shrestha BP, Shrestha D, Tamang S, Thapa S, Tumbahangphe KM, Shrestha JR, Manandhar MK, Manandhar DS: **Implementing a community-based participatory intervention to improve essential newborn care in rural Nepal**. *Trans R Soc Trop Med Hyg* 2003, **97**(1):18-21.
41. Wade A, Osrin D, Shrestha BP, Sen A, Morrison J, Tumbahangphe KM, Manandhar DS, Anthony MdL: **Behaviour change in perinatal care practices among rural women exposed to a women's group intervention in Nepal [ISRCTN31137309]**. *BMC Pregnancy and Childbirth* 2006, **6**(1):20.
42. Srinivasan V, Radhakrishna S, Sudha R, Malathi MV, Jabbar S, Ramakrishnan R, Rao TV: **Randomised controlled field trial of two antenatal care packages in rural south India**. *Indian J Med Res* 1995, **102**:86.
43. Foord F: **Gambia: evaluation of the mobile health care service in West Kiang district**. *World Health Stat Q* 1995, **48**(1):18-22.
44. Mann V, Eble A, Frost C, Premkumar R, Boone P: **Retrospective comparative evaluation of the lasting impact of a community-based primary health care programme on under-5 mortality in villages around Jamkhed, India**. *Bull World Health Organ* 2010, **88**(10):727-736.
45. Turan JM, Tesfagiorghis M, Polan ML: **Evaluation of a community intervention for promotion of safe motherhood in Eritrea**. *J Midwifery Womens Health* 2011, **56**(1):8-17.
46. Xu Z: **China: lowering maternal mortality in Miyun County, Beijing**. *World Health Stat Q* 1995, **48**(1):11-14.
47. Zhang Q: **[Study on health services needs and affecting factors of childbearing-age women of Jingsong Community in Beijing]**. *Zhonghua Yu Fang Yi Xue Za Zhi* 1993, **27**(1):19-21.
48. Alisjahbana A, Williams C, Dharmayanti R, Hermawan D, Kwast BE, Koblinsky M: **An integrated village maternity service to improve referral patterns in a rural area in West-Java**. *Int J Gynaecol Obstet* 1995, **48 Suppl**:S83-94.
49. Bang AT, Bang RA, Baitule SB, Reddy MH, Deshmukh MD: **Effect of home-based neonatal care and management of sepsis on neonatal mortality: field trial in rural India**. *Lancet* 1999, **354**(9194):1955-1961.
50. Bang AT, Bang RA, Baitule SB, Reddy HM, Deshmukh MD: **Management of birth asphyxia in home deliveries in rural Gadchiroli: the effect of two types of birth attendants and of resuscitating with mouth-to-mouth, tube-mask or bagâ€“mask**. *Journal of Perinatology* 2005, **25**:S82-S91.
51. Bang AT, Bang RA, Reddy HM, Deshmukh MD, Baitule SB: **Reduced incidence of neonatal morbidities: effect of home-based neonatal care in rural Gadchiroli, India**. *Journal of Perinatology* 2005, **25**:S51-S61.
52. Bang AT, Reddy HM, Deshmukh MD, Baitule SB, Bang RA: **Neonatal and infant mortality in the ten years (1993 to 2003) of the Gadchiroli field trial: effect of home-based neonatal care**. *Journal of Perinatology* 2005, **25**:S92-S107.
53. Baqui A, Williams EK, Rosecrans AM, Agrawal PK, Ahmed S, Darmstadt GL, Kumar V, Kiran U, Panwar D, Ahuja RC: **Impact of an integrated nutrition and health programme on neonatal mortality in rural northern India**. *Bull World Health Organ* 2008, **86**(10):796-804A.

54. Baqui AH, Rosecrans AM, Williams EK, Agrawal PK, Ahmed S, Darmstadt GL, Kumar V, Kiran U, Panwar D, Ahuja RC: **NGO facilitation of a government community-based maternal and neonatal health programme in rural India: improvements in equity**. *Health Policy and Planning* 2008, **23**(4):234-243.
55. Ronsmans C, Vanneste AM, Chakraborty J, Van Ginneken J: **Decline in maternal mortality in Matlab, Bangladesh: a cautionary tale**. *Lancet* 1997, **350**(9094):1810.
56. Bang AT, Bang RA, Reddy HM: **Home-based neonatal care: summary and applications of the field trial in rural Gadchiroli, India (1993 to 2003)**. *J Perinatol* 2005, **25 Suppl 1**:S108-122.
57. Greenwood AM, Bradley AK, Byass P, Greenwood BM, Snow RW, Bennett S, Hatib-N'Jie AB: **Evaluation of a primary health care programme in The Gambia. I. The impact of trained traditional birth attendants on the outcome of pregnancy**. *J Trop Med Hyg* 1990, **93**(1):58-66.
58. Syed U, Asiruddin S, Helal MSI, Mannan II, Murray J: **Immediate and early postnatal care for mothers and newborns in rural Bangladesh**. *J Health Popul Nutr* 2006, **24**(4):508.
59. Hounton S, SombiÃ© I, Meda N, Bassane B, Byass P, Stanton C, De Brouwere V: **Methods for evaluating effectiveness and costâ€�effectiveness of a Skilled Care Initiative in rural Burkina Faso**. *Tropical Medicine & International Health* 2008, **13**(s1):14-24.
60. Hounton S, Menten J, OuÃ©draogo M, Dubourg D, Meda N, Ronsmans C, Byass P, De Brouwere V: **Effects of a Skilled Care Initiative on pregnancyâ€�related mortality in rural Burkina Faso**. *Tropical Medicine & International Health* 2008, **13**(s1):53-60.
61. Hounton S, Chapman G, Menten J, De Brouwere V, Ensor T, SombiÃ© I, Meda N, Ronsmans C: **Accessibility and utilisation of delivery care within a Skilled Care Initiative in rural Burkina Faso**. *Tropical Medicine & International Health* 2008, **13**(s1):44-52.
62. Newlands D, Yugbareâ€�Belemsaga D, Ternent L, Hounton S, Chapman G: **Assessing the costs and costâ€�effectiveness of a Skilled Care Initiative in rural Burkina Faso**. *Tropical Medicine & International Health* 2008, **13**(s1):61-67.
63. Liu X, Yan H, Wang D: **The evaluation of "Safe Motherhood" program on maternal care utilization in rural western China: a difference in difference approach**. *BMC Public Health* 2010, **10**:566.
64. Fauveau V, Stewart K, Khan SA, Chakraborty J: **Effect on mortality of community-based maternity-care programme in rural Bangladesh**. *Lancet* 1991, **338**(8776):1183-1186.
65. Awasthi S, Srivastava NM, Agarwal GG, Pant S, Ahluwalia TP: **Effect of behaviour change communication on qualified medical care-seeking for sick neonates among urban poor in Lucknow, northern India: a before and after intervention study**. *Trop Med Int Health* 2009, **14**(10):1199-1209.
66. Awasthi S, Verma T, Agarwal M: **Danger signs of neonatal illnesses: perceptions of caregivers and health workers in northern India**. *Bulletin of the World Health Organization* 2006, **84**(10):819-826.
67. Baqui AH, Arifeen SE, Williams EK, Ahmed S, Mannan I, Rahman SM, Begum N, Seraji HR, Winch PJ, Santosham M: **Effectiveness of home-based management of newborn infections by community health workers in rural Bangladesh**. *The Pediatric infectious disease journal* 2009, **28**(4):304.
68. Awasthi S, Srivastava NM, Pant S: **Symptom-specific care-seeking behavior for sick neonates among urban poor in Lucknow, Northern India**. *Journal of Perinatology* 2008, **28**:S69-S75.
69. Agarwal S, Srivastava A: **Social determinants of children's health in urban areas in India**. *Journal of Health Care for the Poor and Underserved* 2009, **20**(4A):68-89.

70. Srivastava NM, Awasthi S, Agarwal GG: **Care-seeking behavior and out-of-pocket expenditure for sick newborns among urban poor in Lucknow, northern India: a prospective follow-up study**. *BMC health services research* 2009, **9**(1):61.
71. Daga SR, Daga AS, Dighole RV, Patil RP: **Anganwadi worker's participation in rural newborn care**. *Indian J Pediatr* 1993, **60**(5):627-630.
72. Emond A, Pollock J, Da Costa N, Maranhao T, Macedo A: **The effectiveness of community-based interventions to improve maternal and infant health in the Northeast of Brazil**. *Rev Panam Salud Publica* 2002, **12**(2):101-110.
73. Fujino Y, Sasaki S, Igarashi K, Tanabe N, Muleya CM, Tambatamba B, Suzuki H: **Improvement in mothers' immediate care-seeking behaviors for children's danger signs through a community-based intervention in Lusaka, Zambia**. *Tohoku J Exp Med* 2009, **217**(1):73-85.
74. Garces A, McClure EM, Hambidge M, Krebs NF, Mazariegos M, Wright LL, Moore J, Carlo WA: **Training traditional birth attendants on the WHO Essential Newborn Care reduces perinatal mortality**. *Acta Obstet Gynecol Scand* 2012, **91**(5):593-597.
75. Saaka M, Galaa S: **Improving the utilization of health and nutrition services: experience from the Catholic Relief Services supported the Development Assistance Programme in Ghana**. *Prim Health Care Res Dev* 2011, **12**(2):145-156.
76. Thassri J, Kala N, Chusintong L, Phongthanasarn J, Boonsrirat S, Jirojwong S: **The development and evaluation of a health education programme for pregnant women in a regional hospital, southern Thailand**. *J Adv Nurs* 2000, **32**(6):1450-1458.
77. O'Rourke K, Howard-Grabman L, Seoane G: **Impact of community organization of women on perinatal outcomes in rural Bolivia**. *Revista Panamericana de Salud PÃºblica* 1998, **3**(1):9-14.
78. Moran AC, Winch PJ, Sultana N, Kalim N, Afzal KM, Koblinsky M, Arifeen SE, Seraji MH, Mannan I, Darmstadt GL *et al*: **Patterns of maternal care seeking behaviours in rural Bangladesh**. *Trop Med Int Health* 2007, **12**(7):823-832.
79. Dongre AR, Deshmukh PR, Garg BS: **Awareness and health care seeking for newborn danger signs among mothers in peri-urban Wardha**. *Indian J Pediatr* 2009, **76**(7):691-693.
80. Deshmukh PR, Dongre AR, Sinha N, Garg BS: **Acute childhood morbidities in rural Wardha: Some epidemiological correlates and health care seeking**. *Indian J Med Sci* 2009, **63**(8):345.
81. Dongre AR, Deshmukh PR, Garg BS: **Childhood morbidity, household practices and health care seeking for sick children in a tribal district of Maharashtra, India**. *Indian J Med Sci* 2010, **64**(1):p7.
82. Dongre AR, Deshmukh PR, Garg BS: **Perceptions and health care seeking about newborn danger signs among mothers in rural Wardha**. *Indian J Pediatr* 2008, **75**(4):325-329.
83. Dongre AR, Deshmukh PR, Garg BS: **A community based approach to improve health care seeking for newborn danger signs in rural Wardha, India**. *Indian J Pediatr* 2009, **76**(1):45-50.
84. Dongre AR, Deshmukh PR, Garg BS: **Health expenditure and care seeking on acute child morbidities in peri-urban Wardha: A prospective study**. *Indian J Pediatr* 2010, **77**(5):503-507.
85. Goudar SS, Dhaded SM, McClure EM, Derman RJ, Patil VD, Mahantshetti NS, Bellad RM, Kodkany B, Moore J, Wright LL: **ENC training reduces perinatal mortality in Karnataka, India**. *Journal of Maternal-Fetal and Neonatal Medicine* 2012, **25**(6):568-574.
86. Matendo R, Engmann C, Ditekemena J, Gado J, Tshefu A, Kinoshita R, McClure E, Moore J, Wallace D, Carlo W: **Reduced perinatal mortality following enhanced training of birth attendants in the Democratic Republic of Congo: a time-dependent effect**. *BMC medicine* 2011, **9**(1):93.

87. Carlo WA, Goudar SS, Jehan I, Chomba E, Tshefu A, Garces A, Sailajanandan P, Althabe F, McClure EM, Derman RJ: **Newborn-care training and perinatal mortality in developing countries**. *N Engl J Med* 2010, **362**(7):614-623.
88. Coskun AM, Karakaya E, Yaser Y: **A safe motherhood education and counselling programme in Istanbul**. *Eur J Contracept Reprod Health Care* 2009, **14**(6):424-436.
89. Hodgins S, McPherson R, Suvedi BK, Shrestha RB, Silwal RC, Ban B, Neupane S, Baqui AH: **Testing a scalable community-based approach to improve maternal and neonatal health in rural Nepal**. *J Perinatol* 2010, **30**(6):388-395.
90. Mushi D, Mpembeni R, Jahn A: **Effectiveness of community based Safe Motherhood promoters in improving the utilization of obstetric care. The case of Mtwara Rural District in Tanzania**. *BMC Pregnancy Childbirth* 2010, **10**:14.
